# Spitting in the wind?—The challenges of RNA sequencing for biomarker discovery from saliva

**DOI:** 10.1007/s00414-023-03100-3

**Published:** 2023-10-17

**Authors:** Annica Gosch, Regine Banemann, Guro Dørum, Cordula Haas, Thorsten Hadrys, Nadescha Haenggi, Galina Kulstein, Jacqueline Neubauer, Cornelius Courts

**Affiliations:** 1 Institute of Legal Medicine, University Hospital of Cologne, Cologne, Germany; 2 Federal Criminal Police Office, Forensic Science Institute, Wiesbaden, Germany; 3 Zurich Institute of Forensic Medicine, University of Zurich, Zurich, Switzerland; 4 State Criminal Police Office, Forensic Science Institute, Munich, Germany

**Keywords:** Forensic RNA analysis, Saliva, Massive parallel sequencing

## Abstract

Forensic trace contextualization, i.e., assessing information beyond who deposited a biological stain, has become an issue of great and steadily growing importance in forensic genetic casework and research. The human transcriptome encodes a wide variety of information and thus has received increasing interest for the identification of biomarkers for different aspects of forensic trace contextualization over the past years. Massively parallel sequencing of reverse-transcribed RNA (“RNA sequencing”) has emerged as the gold standard technology to characterize the transcriptome in its entirety and identify RNA markers showing significant expression differences not only between different forensically relevant body fluids but also within a single body fluid between forensically relevant conditions of interest. Here, we analyze the quality and composition of four RNA sequencing datasets (whole transcriptome as well as miRNA sequencing) from two different research projects (the RNAgE project and the TrACES project), aiming at identifying contextualizing forensic biomarker from the forensically relevant body fluid saliva. We describe and characterize challenges of RNA sequencing of saliva samples arising from the presence of oral bacteria, the heterogeneity of sample composition, and the confounding factor of degradation. Based on these observations, we formulate recommendations that might help to improve RNA biomarker discovery from the challenging but forensically relevant body fluid saliva.

**Supplementary Information:**

The online version contains supplementary material available at 10.1007/s00414-023-03100-3.

## Introduction

The main aim of forensic molecular biological analysis is the individualization of a trace, i.e., unequivocally linking a biological trace to its donor, which is commonly performed via DNA-based STR profiling. Apart from and complementary to that, the contextualization of traces has become an issue of great and steadily growing importance. If the donor of a trace is not contested in a criminal court case, it can be crucial in the reconstruction of the course of events to contextualize the trace, meaning to explain, based on physical evidence, by which activity, how long ago, at which time of day, as part of which body fluid or organ tissue, etc., and the trace in question has been deposited.

Because of the high and complex information content of the transcriptome [1] represented by its differential and dynamically changing composition, the analysis of RNA readily lends itself to the assessment of several forensic contextual aspects. Among these, the identification of body fluids via gene expression analysis [2] is routinely applied in forensic casework in different laboratories [3]. Besides, several research projects assess the potential of transcriptomic analysis for assessing further aspects of forensic relevance, such as time since trace deposition [4], post-mortem interval estimation [5], wound age [6], the biological age of the donor [7], and time of day of deposition [8]. Since 2009, also microRNA (miRNA), a small, non-coding regulatory type of RNA, about 18–25 nt in length, is being investigated in the context of its forensic potential [9] and ongoing research into a wide array of forensic applications of miRNA analysis has covered a lot of ground up to this point [10, 11].

Frequently, the selection of mRNA and miRNA candidates whose differential expression and/or degradation state [12] informs on a particular aspect of forensic interest will be performed by perusing (not necessarily forensic) literature and testing previously identified markers for their informative value in the setup of interest (for example performed in [8, 13]). However, previously published markers may not always be ideal or even available to answer the question of forensic interest at hand, and thus, traditional statistical and machine learning algorithms based upon raw data generated by massively parallel sequencing (MPS) of whole transcriptomes [14] and miRNomes [15] are increasingly being used for unsupervised marker identification.

Saliva is a body fluid regularly encountered in several criminal contexts with cases of sexual assault (e.g., licked, bitten, or spat on body parts, oral rape) being of particular impact. Therefore, the reliable and sensitive detection, identification, and contextualization of saliva in (mixed) trace material is very desirable and can be crucial in the assessment of the trace’s weight of evidence. Consequently, analysis of salivary RNA from forensic samples is well represented in the literature [16–20], with a particular focus on body fluid identification. Additionally, saliva is often included in marker identification studies for other trace contextualization aspects [4, 21].

Within this article, the authors—a European consortium of forensic RNA researchers working on different aspects of RNA-based trace contextualization—present RNA sequencing data from different sets of salivary samples and experimental setups and discuss the challenges associated with whole transcriptome sequencing of saliva samples.

## Material and methods

Evaluations are based on four different RNA sequencing datasets from saliva samples obtained in two different forensic genetic research projects: RNA-based Age Estimation (RNAgE) and Transcriptomic Analysis for the Contextualization of Evidential Stains (TrACES). Both research projects have the aim of identifying RNA markers for a forensically relevant aspect of trace contextualization. The first project aimed at correlating the transcriptome with the sample donor’s age (hereafter referred to as “RNAgE” project), while the second project explored potential correlations of the transcriptome with the time of day of sample collection (hereafter referred to as “TrACES” project). In both research projects, donors provided samples of blood as well as saliva. Results for the transcriptomic analysis of blood samples are reported separately for the “RNAgE” (manuscript under preparation) and the “Traces” [22] projects.

In both projects, salivary transcriptomes were analyzed using both whole transcriptome (WT) and microRNA (miRNA) sequencing, resulting in a total of four datasets (Table 1).

**Table 1:** Sample collection and processing information for the four different saliva sample sets

Sample set abbreviation	RNAgE—WT	TrACES—WT	RNAgE—miRNA	TrACES—miRNA
Aspect of contextualization	Donor age	Time of day	Donor age	Time of day
Targeted RNA type	Total RNA (excluding rRNA)	Total RNA (excluding rRNA)	miRNA	miRNA
Number of Samples and Individuals	67 individuals (39 females, 28 males; 8–77 years); 1 sample per individual	3 individuals (2 males, 1 female, 25–31 years), 8 samples per individual (8 time points: 8 AM, 11 AM, 2 PM, 5 PM, 8 PM, 11 PM, 2 AM, 5 AM); 24 samples in total	85 individuals (56 females, 29 males; 0–96 years); 1 sample per individual	10 individuals (5 males, 5 females; 19–31 years), 8 samples per individual (8 time points: 8 AM, 11 AM, 2 PM, 5 PM, 8 PM, 11 PM, 2 AM, 5 AM); 80 samples in total**
Sample collection	Cotton swab (Forensix sample collection system, ThermoFisher Scientific) soaked in liquid saliva* (after spitting)	Buccal mucosa collected on a cotton swab (Sarstedt)*	Cotton swab (Forensix sample collection system, ThermoFisher Scientific) soaked in liquid saliva* (after spitting)	Buccal mucosa collected on a cotton swab (Sarstedt)*
Sample stabilization/storage	Dried and stored at RT for up to 11 days	Stabilized in DNA/RNA shield stabilization solution (Zymo Research) and stored at − 80 °C	Dried and stored at RT for up to 11 days	Stabilized in DNA/RNA shield stabilization solution (Zymo Research) and stored at − 80 °C
RNA extraction	ReliaPrep RNA Miniprep System, (Promega)	mirVana miRNA extraction kit (total RNA protocol), (Thermo Fisher Scientific)	miRNeasy mini Kit (QIAGEN)	mirVana miRNA extraction kit (total RNA protocol), (Thermo Fisher Scientific)
DNAse treatment	TURBO DNase free Kit, Thermo Fisher Scientific)	TURBO DNase free Kit, Thermo Fisher Scientific)	TURBO DNase free Kit, Thermo Fisher Scientific)	TURBO DNase free Kit, Thermo Fisher Scientific)
RNA quantification	QuantiFluor RNA System, HS assay, (Promega)	QuantiFluor RNA System, HS assay, (Promega)	QuantiFluor RNA System, HS assay, (Promega)	QuantiFluor RNA System, HS assay, (Promega)
Quality control	Not assessed	RNA Pico Chip Kit on a 2100 Bioanalyzer Instrument (Agilent Technologies)	Not assessed	RNA Pico Chip Kit on a 2100 Bioanalyzer Instrument (Agilent Technologies)
Library preparation	TRIO RNA Seq Library Preparation Kit, no rRNA depletion (Tecan)	Stranded total RNA with Ribo-Zero Plus protocol, Ribo-Zero Plus rRNA depletion kit (Illumina), performed at the Competence Centre for Genomic Analyses in Kiel, Germany	NEBNext multiplex small RNA library Prep Set for Illumina (NEB)	NextFlex Small RNA library preparation kit (Bioo Scientific) performed at the Competence Centre for Genomic Analyses in Kiel, Germany
Sequencing	NovaSeq 6000 platform, Illumina, S1 flowcell (100 nt, single end, 15 Mio. Reads/sample), performed at the Functional Genomics Center Zurich (FGCZ), Switzerland	NovaSeq 6000 platform, Illumina, S4 flowcell, (2 × 50 bp, paired-end, 30 Mio. Reads/sample), performed at the Competence Centre for Genomic Analyses in Kiel, Germany	NextSeq 500 platform, Illumina, High output flowcell, (75 nt, single end, 13 Mio. Reads/sample), performed at the Functional Genomics Center Zurich (FGCZ), Switzerland	NovaSeq 6000 platform, Illumina, S1 flowcell, (2 × 50 bp, paired-end, 16 Mio. Reads/sample), performed at the Competence Centre for Genomic Analyses in Kiel, Germany

*Donors refrained from eating and drinking (except water) for ½ h prior to sampling

**In the TrACES – miRNA dataset, library preparation failed for two samples, thus only 78 out of 80 samples were submitted to sequencing

Details on sample collection and sample processing are provided in Bioinformatic processing of datasets was performed as described in Table 2.

**Table 2:** Bioinformatic processing information of RNA sequencing data for the four different saliva sample sets. All processing steps for the WT datasets were performed on the free public European Galaxy server UseGalaxy.eu [23]

Sample set abbreviation	RNAgE—WT	TrACES—WT	RNAgE—miRNA	TrACES—miRNA
Preprocessing	Trimmomatic (Adapter trimming, Removal of 5 bases from the start of the read, keeping reads of a minimal length of 20 nt and a minimum average quality of 10) [24]	Trim Galore! (Adapter trimming, Removal of 1 base from the start of the read, removal of low-quality ends from reads, keeping reads of a minimal length of 20 nt) [25]	sRNAbench (default settings for the NEBnext protocol) [26, 27]	sRNAbench (default settings for the Bioo Scientific Nextflex (v2,v3) protocol), processing of forward strand reads (R1) only [26, 27]
Mapping	STAR mapping algorithm, single-end reads, using the GenCode primary assembly reference genome and primary assembly comprehensive gene annotation (GRCh38.p13, Release 43) [28, 29]	STAR mapping algorithm, paired-end reads, using the GenCode primary assembly reference genome and primary assembly comprehensive gene annotation (GRCh38.p13, Release 43) [28, 29]	sRNAbench (default settings for the NEBnext protocol, annotation reference database: miRBase release 22.1, species: *Homo sapiens*) [26, 27, 30]	sRNAbench (default settings for the Bioo Scientific Nextflex (v2,v3) protocol, annotation reference database: miRBase release 22.1, species: *Homo sapiens*), processing of R1 reads only [26, 27, 30]
Assessment of ribosomal RNA (rRNA) content	SortMeRNA on trimmed reads, rRNA databases: 2.1b-rfam-5 s-database-id98 2.1b-silva-arc-23 s-id98 2.1b-silva-euk-28 s-id98 2.1b-silva-bac-23 s-id98 2.1b-silva-euk-18 s-id95 2.1b-silva-bac-16 s-id90 2.1b-rfam-5.8 s-database-id98 2.1b-silva-arc-16 s-id9 [31–33]*	SortMeRNA on trimmed reads, rRNA databases: 2.1b-rfam-5 s-database-id98 2.1b-silva-arc-23 s-id98 2.1b-silva-euk-28 s-id98 2.1b-silva-bac-23 s-id98 2.1b-silva-euk-18 s-id95 2.1b-silva-bac-16 s-id90 2.1b-rfam-5.8 s-database-id98 2.1b-silva-arc-16 s-id9 [31–33]*	sRNAbench (default settings for the NEBnext protocol) [26, 27]	sRNAbench (default settings for the Bioo Scientific Nextflex (v2,v3) protocol), processing of R1 reads only [26, 27]
Assessment of bacterial RNA content	Kraken2 on trimmed reads, Bracken, Krakentools, Krona [34–38]	Kraken2 on trimmed reads, Bracken, Krakentools, Krona [34–38]	sRNAblast on unassigned reads from sRNAbench analysis [26, 27]	sRNAblast on unassigned reads from sRNAbench analysis [26, 27]
Assessment of on-target read counts	“reads per gene” output from the STAR mapping algorithm [28]	“reads per gene” output from the STAR mapping algorithm [28]	sRNAbench (default settings for the NEBnext protocol), SA read counts provided in the mature_sense_SA output file [26, 27]	sRNAbench (default settings for the Bioo Scientific Nextflex (v2,v3) protocol), SA read counts provided in the mature_sense_SA output file [26, 27]

*Reads mapping to human mitochondrial rRNA (ENSG00000210082.2 and ENSG00000211459.2) were additionally counted as rRNA reads and excluded from the on-target read count table

Table 1. All protocols were performed according to the manufacturers’ instructions.

Bioinformatic processing of datasets was performed as described in Table 2.

## Results

**Fig. 1:**
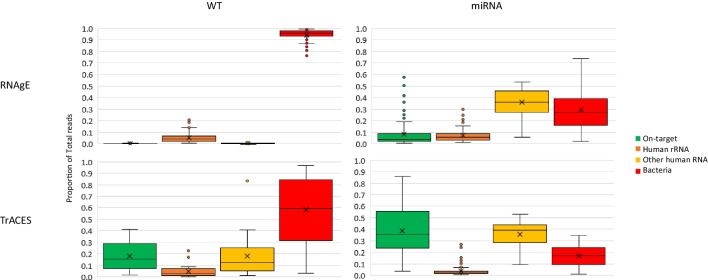
Distribution of sequencing reads in four RNA sequencing datasets from saliva samples. Boxplots *indicate the distribution of the proportion of sequencing reads over n* = *67, 24, 85, 78 (RNAgE-WT, TrACES-WT, RNAgE-miRNA, and* TrACES-miRNA datasets respectively) samples. The boxplots indicate the median and interquartile ranges (IQR), whiskers indicate the minimum and maximum values (within 1.5*IQR)*, and outliers (*> 1.5*IQR) are indicated by individual dots. Additionally, the average value is marked by a cross. WT, whole transcriptome*.* “On-target” reads are defined as reads mapping to exonic regions of genes in the WT datasets (cf. Supp. Table 1) and reads mapping to miRbase (sense) in the miRNA datasets (cf. Supp. Table 2). “Other human RNA” reads are assigned to the human transcriptome but did not map to the target RNA type or human rRNA (i.e., ambiguously mapped reads, multi-mapping reads, intronic and intergenic reads for the WT datasets), and reads assigned to RNA types other than miRNA and rRNA (e.g., small nuclear RNA (snRNA), small nucleolar RNA (snoRNA), mRNA, long-non coding RNA (lncRNA) in miRNA datasets)

In two different research projects, RNA sequencing was performed to identify biomarkers (human RNA transcripts or human miRNA) showing differential expression either between individuals of different ages (“RNAgE” project) or between samples deposited at different times of the day (“TrACES” project). An evaluation of the distribution of sequencing reads in the four different datasets from human saliva samples showed that only a low percentage of reads mapped to the actual RNA species of interest (Fig. 1). The percentage of human on-target reads was lowest in the “RNAgE-WT” dataset (average: 0.1%, range: 0.007–1.1%) and highest in the “TrACES-miRNA” dataset (average: 38.4%, range: 3.8–86.2%). For a better understanding of the reasons for these low on-target read count percentages, we performed an in-depth analysis of the read distribution of the four sequencing datasets (Fig. 1, Supplementary Tables 1 and 2).

### Non-human RNA

Taxonomic classification of reads revealed that the largest proportion of reads in both WT datasets is of bacterial origin (Fig. 1). Sequences from a diverse set of microbial species were detected (Supp. File 1 and 2), most of which are commonly encountered in the human oral cavity [39]. The percentage of bacterial reads was consistently > 75% in the “RNAgE-WT” dataset and thus accounted for the majority of reads in every sample of this dataset. The “TrACES-WT” dataset contained lower percentages of bacterial reads (average: 58%, range: 3–97%) with large variability between individuals as well as between samples from the same individual taken at different time points of the day (Supp. Table 1).

In both miRNA datasets, the percentage of microbial reads was lower compared to the whole transcriptome sequencing dataset from the corresponding research project (average < 30% in both datasets, Supp. Table 2).

### Human RNA

In the “RNAgE-WT” dataset, the majority of human reads were attributed to ribosomal RNA (rRNA) (average: 94.1%, Fig. 1, Supp. Table 1). Thus, among the already low proportion of reads mapping to the human transcriptome, the proportion of reads mapping to regions of interest (i.e., genes) was also low (average: 2.5%, Supp. Table 1). In comparison, the percentage of reads mapping to rRNA was considerably reduced in the “TrACES” dataset (average: 11%, Supp. Table 1). In the “RNAgE” dataset, most of the ribosomal reads were from mitochondrial rRNA (average mitochondrial to nuclear rRNA ratio: 12.7, range: 1.5–83.0), whereas in the “TrACES” dataset, a higher proportion of nuclear rRNA reads was observed (average ratio: 0.1, range: 0.02–0.3, Supp. Table 1). In the “TrACES” dataset, the percentage of human reads mapping to regions of interest was on average 48.4% (Supp. Table 1). Besides the reads mapping to genes, a relevant proportion (average: 27%) also mapped to intronic or intergenic regions of the transcriptome (assigned as “no Feature” by the STAR mapping algorithm, Supp. Table 1).

In the miRNA sequencing datasets, rRNAs made up a relatively small proportion of the total reads of human origin (average of 13.6% and 4.7% in the “RNAgE” and “TrACES” datasets respectively, Supp. Table 2). However, significant proportions of human reads were attributed to other (small) RNA species, resulting in average “on target”-miRNA reads of 13.7 and 46.9% of total human reads in the “RNAgE” and “TrACES” datasets, respectively (Supp. Table 2).

### “Useful reads” for biomarker discovery

To apply algorithms for the identification of biomarkers using differential gene expression analysis, potential RNA markers must be reliability detected and quantified. Thus, markers with very low read counts are commonly excluded from datasets prior to the application of differential gene expression algorithms [40].

**Fig. 2:**
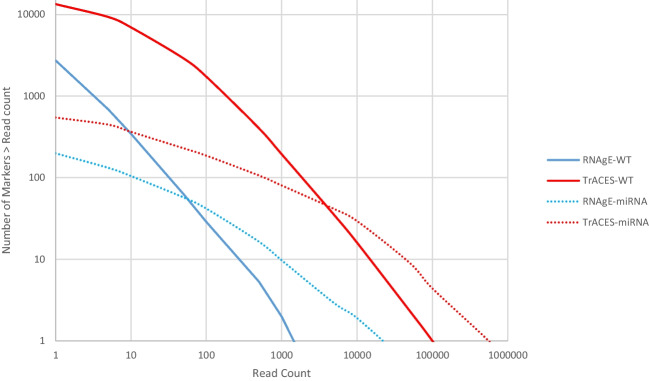
Number of markers above a read count in each of the four RNA sequencing datasets. The plot indicates average values over *n* = 67, 24, 85, s78 (RNAgE-WT, TrACES-WT, RNAgE-miRNA, and TrACES-miRNA datasets respectively) samples

The number of RNA markers detected above a certain read count in each of the four sequencing datasets is plotted in Fig. 2. (Note: Data is shown as raw read counts in Fig. 2. For the purpose of performing differential gene expression analysis within datasets, these read counts would have to be normalized. However, as in this step we were interested in the number of markers whose expression can reliably be quantified (i.e., can be differentiated from noise), raw read counts were considered here.)

It is evident that in each of the four datasets, the number of RNA markers that can be reliably quantified (and thus may be eligible for biostatistical analyses for marker discovery) is very limited. In the “RNAgE-WT” dataset, an average of 29 markers (range: 0–264) exceeded an absolute read count threshold of 100. In the “TrACES –WT” dataset, a higher number of markers reached this threshold (average: 1731, range: 130–4020); however, this number remains low compared to a whole transcriptome sequencing dataset of human whole blood that had been processed in a similar way in the “TrACES” project (average: 10,669, range: 8602–16,483 in a total of 80 samples from ten individuals, data not shown).

The number of miRNA markers reaching an absolute read count threshold of 100 was 42 (4–156) and 186 (49–273) in the “RNAgE” and “TrACES” datasets, respectively.

## Discussion

We analyzed four different RNA sequencing datasets from saliva samples originating from two different research projects. In each of these datasets, data analysis proved to be challenging due to a large heterogeneity between samples and consistently low percentages of read counts aligning with the RNA targets of interest. Nevertheless, differences were observed between datasets that were analyzed under different conditions, indicating that some of the challenges associated with RNA sequencing of saliva samples may be addressed by adjusting the analysis procedures. It needs to be emphasized that this study was not designed to systematically assess how individual aspects within each of the different workflows impacted the total outcome. Nonetheless, the observations reported herein allow for some conclusions that merit consideration for future biomarker discovery studies for forensic (and non-forensic) purposes.

Based on the in-depth analysis of four RNA sequencing datasets (whole transcriptome and miRNome) from two different forensic research projects, we identified three factors contributing to the challenges of RNA sequencing from saliva samples.

### Non-human RNA content

Whole transcriptome analyses showed that a large proportion of the RNA present in the salivary samples was of bacterial origin, which is consistent with previous studies [41–43]. For example, Ostheim et al. describe that the bacterial RNA content in human saliva is on average 1145 times higher than the human RNA content (based on 18S/16S rRNA ratio measurements) [42].

In comparison to the “RNAgE-WT” dataset, the bacterial RNA content was reduced in the “TrACES” dataset. The stabilization of buccal mucosa samples right after collection (possibly preventing bacterial growth), as well as the use of the Ribo-Zero Plus rRNA depletion kit (depleting not only human but also gram-negative (*Escherichia coli*) and gram-positive (*Bacillus subtilis*) bacterial 5S, 16S, and 23S rRNA sequences), might have contributed to the reduction of bacterial reads in this dataset.

As bacteria are naturally present in human saliva, it is hardly possible to select and sequence only the human component of the salivary transcriptome. Biomedical studies often analyze cell-free saliva (obtained by centrifugation) rather than whole saliva, as it has been shown to contain a lower proportion of bacterial RNA than whole saliva [43]. However, this approach would not be applicable to forensic saliva stains as these are usually dried (e.g., on surfaces at a crime scene) hindering a clear separation into cellular and cell-free fractions.

In another previous study, the authors observed higher percentages of reads mapping to the human transcriptome when enriching for poly-A-tailed RNAs (rather than depleting rRNAs), as polyadenylation of mRNAs is unique to eukaryotic cells [44]. However, the authors also remark that this approach restricts biomarker discovery to polyadenylated mRNA, whereas non-polyadenylated transcripts (such as non-coding RNAs) will not be detectable [44]. Additionally, it has been experimentally proven that more comprehensive and reliable results can be obtained from low-quality/degraded samples with rRNA-depletion-based library preparation methods as compared to oligo(dT)-enrichment-based methods [45], which is why this approach is usually recommended for samples expected to show degradation to some extent (cf. “Heterogeneity of sample composition”).

Thus, the presence of oral bacteria in saliva has to be accepted and needs to be accounted for when processing salivary samples: Based on our observations, we recommend adjusting the sequencing depth to account for the high percentage of reads expected to map to bacterial rather than human RNA. Additionally, an adjustment of bioinformatics processing workflows was recommended by Kaczor-Urbanowicz et al. Reads mapping to bacterial genomes should be filtered out in a first bioinformatics processing step, prior to analyzing the reads mapping to the human transcriptome [46].

Alternatively, the oral microbiome could represent a target for the discovery of (forensic) biomarkers: In biomedical studies, the composition of the oral microbiome has been associated with a number of oral as well as non-oral diseases (including periodontitis, cardiovascular disease, and pneumonia, e.g., summarized in [47, 48]). In a forensic context, microbial signatures have very early been suggested for analysis in addition to human transcripts for the differentiation of forensically relevant body fluids [49–51]. More recently, it has also been shown that changes in the composition of microbial transcripts could be used as a biomarker to analyze time since deposition of forensically relevant body fluids (including saliva) [41]. Hence, the salivary microbiome could be eligible for biomarker discovery for forensic trace contextualization by considering not only its composition on a species-level but also individual differentially expressed bacterial transcripts.

A second alternative solution would be to target the miRNome rather than the whole transcriptome. miRNAs are small, regulatory RNAs present only in eukaryotes and thus naturally absent from bacterial transcriptomes [52]. Indeed, in both research projects, the percentage of reads assigned to the human transcriptome was higher in the miRNA sequencing datasets as compared to the WT sequencing datasets.

Nonetheless, bacterial reads were still present in the majority of the samples. In both library preparation procedures performed in this study, small RNAs are enriched by selecting RNA molecules of a defined fragment length (corresponding to the combined length of small RNAs of interest and adjoined adapters). Thus, fragmented bacterial RNA of the same length will also be included in the resulting small RNA sequencing libraries.

As described for whole transcriptome sequencing of saliva samples, the issue of bacterial reads in miRNA sequencing can be addressed and might (at least partially) be resolved by the choice of sample preparation protocol, adjustment of sequencing depth to account for off-target reads as well as modified bioinformatic processing protocols [46].

### Complexity of the human transcriptome

In the “RNAgE” WT dataset, the majority of reads mapping to the human transcriptome was determined to be rRNA. rRNAs are known to make up a considerable proportion (≥ 80%) of the human transcriptome [45, 53].

Therefore, it is common to perform some sort of enrichment of target RNA species (either enrichment of poly-A-tailed (mostly) mRNA (“poly-A-enrichment”) or selective depletion of rRNA (“rRNA depletion”) [54]. When deciding on an RNA sequencing strategy for the “RNAgE” project, it was taken into consideration that for the TRIO RNA Seq Library Preparation kit, the rRNA depletion step had previously been observed to have a negative influence on the sequencing quality and on downstream analyses [55]. Additionally, studies suggest that rRNAs may carry age-relevant information [56, 57]. Thus, the rRNA depletion step was omitted in the “RNAgE” project. Therefore, it may plausibly be assumed that the relevant reduction of rRNA reads in the “TrACES” dataset as compared to the “RNAgE” dataset can be explained by the inclusion of the rRNA depletion step during library preparation of samples in this study.

Notably, we observed a large proportion of rRNA reads mapping to mitochondrial RNA in the “RNAgE” dataset. As mitochondria have evolutionary originated from incorporated prokaryotes [58], it may be hypothesized that a proportion of these reads was of true bacterial origin and incorrectly mapped to the human mitochondrial rRNA (mt-rRNA). However, as the absolute reads mapped to mt-rRNA reads did not relevantly decrease when STAR mapping was performed on reads not aligned to rRNA in the SortMeRNA step (data not shown), a true human mitochondrial origin is more likely. In single-cell-RNA sequencing, high proportions of mitochondrial reads are considered indicative of damaged cells (and corresponding cells are usually excluded from analysis) [59, 60]. Our observations might thus suggest a high proportion of damaged cells in human saliva (which is to be expected). However, to the best of our knowledge, no other studies report similarly high proportions of mt-rRNA in whole transcriptome sequencing datasets from saliva (available studies either performed rRNA depletion or did not specifically report rRNA read proportions [43, 46, 61, 62]). The association between high mt-rRNA reads and cell damage in whole transcriptome sequencing datasets thus remains speculative and would have to be experimentally assessed in future studies.

Besides exonic reads, relevant percentages of reads mapping to intronic and intergenic regions were observed in both WT datasets. It has previously been reported that high percentages of intronic and intergenic reads (e.g., representing non-coding transcripts or nascent mRNAs) are commonly seen in rRNA-depleted whole transcriptome sequencing libraries (as opposed to poly-A-enriched sequencing libraries in which all transcripts lacking poly-A-tails would be excluded) [63, 64]. In comparison to poly-A-enriched libraries, rRNA depleted libraries capture information encoded in the entirety of the transcriptome and therefore require a higher sequencing depth to achieve a similar exonic coverage [63].

Compared to the WT datasets, miRNA sequencing resulted in higher percentages of on-target read counts. The higher proportion of human reads mapping to miRNAs in the “TrACES” dataset compared to the “RNAgE” dataset might be attributed to the differences in sampling procedures (liquid saliva dried on cotton swabs vs. stabilized buccal swabs). Sullivan et al. observed significant differences in miRNome compositions between whole saliva samples stored with and without RNA stabilizer (with significantly higher miRNA read counts for samples stored in stabilizer). Moreover, they found differences attributable to the collection method (with significantly higher miRNA read counts for samples collected by swabbing compared to samples collected by expectoration), and highlight the relevance of consistent sample collection and storage procedures within studies [65].

Besides, the higher proportion of miRNAs reads in the “TrACES” dataset might also be caused by the use of a different library preparation procedure. Comparative evaluations of small RNA library preparation procedures have repeatedly reported higher proportions of miRNA reads and larger numbers of miRNAs detected when using the Bioo Scientific NextFlex Small RNA-Seq library preparation kit as compared to the NEBNext Small RNA Library Prep Kit [66, 67].

miRNA sequencing libraries also showed a large complexity comprising a variety of different RNA types including mRNAs, rRNAs, lncRNAs, snRNAs, snoRNAs, and other small RNAs (not specifically targeted by the miRNA mapping algorithm applied in this study [26, 27]). Types of small RNA other than miRNA, e.g., PIWI-interacting RNA (piRNA), snRNA, and snoRNA have already been reported as biomarkers in previous forensic studies [68, 69] and could be possibly included in future marker identification studies as well.

### Heterogeneity of sample composition

The transcriptomic composition in the datasets from two different studies showed a large heterogeneity, both between as well as within studies. Differences between sample sets partially arise from different technologies (as discussed above) but can also be attributable to the different sample types (liquid saliva samples in the “RNAgE” and buccal swab samples in the “TrACES” dataset):

In forensic as well as biomedical studies, both liquid saliva and swabbed samples of the buccal mucosa (“buccal swabs”) are used to study the body fluid “saliva.” However, the two sample types have been shown to possess a markedly different biological composition [70]. Liquid saliva is a complex mixture of fluids secreted from various glands in the oral cavity. It is composed of > 99% water with a pH between 6 and 7 under normal conditions, and contains a large variety of electrolytes as well as macromolecules, such as mucins, enzymes, and immunoglobulins [71]. The cellular content of liquid saliva is low and mainly consists of leukocytes, erythrocytes, and epithelial cells shed from the oral mucosa [72]. A study analyzing microscopy slides with saliva observed that 47.3% (± 6.2) of the cells found in liquid saliva from adult study participants were of epithelial origin, compared to 83.4% (± 6.8) in the same participants’ buccal swabs [70]. As cellular heterogeneity impacts heterogeneity in the transcriptome, it is not recommended to mix liquid saliva and buccal swab samples in biomarker discovery studies.

In forensic casework, salivary samples may be recovered in a variety of different contexts, deriving from kissing, licking, spitting, drooling, chewing, biting, or speaking. Both liquid saliva and buccal swabs may represent only a subset of these forensically relevant salivary sample types [73] and it thus remains open for discussion which sample type is best suited for forensic biomarker discovery studies.

By microscopic evaluation, both liquid saliva and buccal swab samples showed large inter-individual differences in cell type composition (with liquid saliva showing a higher variability than buccal swabs) [70]. This is consistent with larger inter- as well as intra-individual variability observed in the datasets presented in this study.

Apart from the previously described cellular RNA, cell-free RNA (cfRNA) has been reported to contribute to the transcriptome of human saliva [74, 75]. cfRNAs may originate from dead or damaged cells [69, 71], but have also been shown to reside within exosomes secreted by cells in human saliva [76]. For miRNAs, it has even been suggested that exosomes are the main source of this RNA species in human saliva [75].

The heterogeneity of the salivary transcriptome’s composition is further increased by degradation. Previous studies have reported both full-length and partially degraded RNA molecules in human saliva [76, 77]. Studies analyzing the time-wise stability of the transcriptome in different body fluids observed that the salivary transcriptome showed low integrity even right after sample deposition, and salivary transcripts degraded more rapidly compared to other body fluids (blood, semen, and vaginal secretions) [4, 12, 78]. Conditions in the oral cavity (warmth, moisture, presence of ribonucleases) are assumed to promote salivary RNA degradation, but it has also been shown that exogenously introduced mRNA degrades more rapidly under these conditions than endogenous salivary RNA, suggesting that the salivary RNA might be (partially) protected [77].

Previous studies have measured the extent of overall as well as transcript-wise degradation based on the assessment of the sequencing coverage along the transcript, with degraded transcripts showing increased coverage at their 3’ ends [79]. However, this approach enables quantification of the extent of degradation only for sequencing libraries that have been prepared using the poly-A-enrichment strategy, as it selects the 3’-fragments of transcripts, whereas a 3’ bias is not expected to be observed for degraded samples after selective depletion of rRNAs [80].

While we are thus unable to exactly quantify and compare the amount of degradation in the samples analyzed in this study, it may reasonably be assumed based on observations from previous studies that RNA from human saliva is degraded to a certain extent, and that the stochastic phenomenon of degradation increases heterogeneity within sample sets.

Due to their short length, miRNAs are assumed to be less prone to degradation, and previous studies have indeed observed these small RNAs to be more stable than mRNA transcripts [81–83]. Hence, despite our lack of knowledge of the true extent of miRNA degradation in our datasets, there is sufficient ground to assume that the increased quality and on-target read counts in our miRNA compared to the WT datasets may partly be attributable to the lower impact of degradation on miRNAs as compared to longer transcripts.

## Conclusion

In summary, our analysis of four different RNA sequencing datasets from two different forensic research projects indicates that biomarker discovery from saliva samples through RNA sequencing is challenging. This can be attributed to a multitude of factors that decrease the proportion of sequencing reads suitable for biomarker discovery, and at the same time increase between-sample heterogeneity. This includes the presence of oral bacteria, the heterogeneity of cellular and cell-free RNA, and the confounding factor of degradation.

It is important to note that our study was not explicitly designed to assess the impact of individual impact of factors such as sample collection procedures, stabilization reagent, or library preparation methods. As a result specific recommendations for an optimal sample processing protocol cannot be given based on our observations. However, our results in combination with discussed outcomes of previous studies may be helpful to inform decisions and designs for future biomarker discovery studies.

To address the issue of low read counts for the RNAs of interest, we recommend adjusting the sequencing depth or undertaking measures to enrich for the RNA type of interest. This may include the control for microbial growth by sample stabilization after collection, selective depletion of (bacterial and) human rRNA, or selective enrichment of poly-A-tailed RNA.

Besides human mRNA, other biomarkers may be more suitable for saliva samples: The high bacterial load might be exploited in metagenomic/metatranscriptomic analyses. Alternatively, small RNAs, which are less prone to degradation and whose sequencing results are less impacted by the presence of bacteria, might also be promising targets for biomarker discovery studies.

In conclusion, careful experimental planning that should account for the challenges associated with the important, but difficult-to-tackle body fluid “saliva,” and adjusting for individual research aims, will be necessary to successfully parse the salivary transcriptome for biomarkers that can potentially contextualize forensically relevant saliva stains.

### Supplementary Information

Below is the link to the electronic supplementary material.

Supplementary file 1: Taxonomic classification of sequencing reads assessed by Kraken2-Bracken anaylsis and visualized by Krona [32-36], combined Analysis of 67 samples from 67 individuals from the “RNAgE” WT dataset (HTML 1095 KB)

Supplementary file 2: Taxonomic classification of sequencing reads assessed by Kraken2-Bracken anaylsis and visualized by Krona [32-36], combined Analysis of 24 samples from 3 individuals from the “TrACES” WT dataset (HTML 957 KB)

Supplementary file 3 (XLSX 52 KB)

## Declarations

In the “RNAgE” project, all sample donors provided written informed consent. The study protocols were reviewed and approved by the CEBES review board at the University of Zurich (case number 2021-11e).

In the “TrACES” project, all sample donors provided written informed consent. The study protocols were reviewed and approved by the ethics committee of the University Hospital Schleswig–Holstein.
