# Supplementary material for: Spitting in the wind?—The challenges of RNA sequencing for biomarker discovery from saliva
Source: Int J Legal Med. 2023 Oct 17;138(2):401–12. doi: 10.1007/s00414-023-03100-3 (PMC10861700; doi:10.1007/s00414-023-03100-3)
Supplement: Supplementary file 1 — Taxonomic classification of sequencing reads assessed by Kraken2-Bracken anaylsis and visualized by Krona [32-36], combined Analysis of 67 samples from 67 individuals from the “RNAgE” WT dataset (HTML 1095 KB) [file 414_2023_3100_MOESM1_ESM.html]

Javascript must be enabled to view this page.

magnitude
magnitudeUnassigned

1913576682

0
5587

0
285

285
0

0
16

16
0

16
0

16

30
0

0
30

30
0

30

0
239

0
239

106
0

106

0
117

117

16
0

16

0
1640

1640
0

1640

3361
0

67
0

67
0

67
0

67
0

50

17

1581
0

1581
0

1539
0

33
0

14

19

1206
0

977

129

49

35

16

0
300

87

213

0
42

0
42

42

78
0

78
0

0
78

0
10

10

0
13

13

0
55

37

18

81
0

61
0

0
61

0
15

15

0
46

23

23

20
0

0
20

0
20

20

0
646

0
646

0
565

0
565

53

44

468

81
0

81
0

65

16

0
908

244
0

0
244

0
244

244

0
553

438
0

438
0

23

415

115
0

25
0

25

0
16

16

0
74

16

48

10

0
111

0
111

0
111

111

0
16

0
16

16
0

16
0

16
0

16

78
0

78
0

78

207
0

65
0

65
0

65
0

0
65

65

142
0

142
0

0
30

30

112
0

74

14

24

107950075
0

0
107950075

0
107950075

107950075
0

107950075
0

0
107950075

107950075
0

107950075

4818147
0

4815679
0

0
4815679

0
4815679

0
1584

11

1486
0

983

503

87

4113
0

16

0
34

34

20

25

3685

14

0
20

20

14
0

14

14
0

14

260

0

11
0

11

4809959
0

602

1867

2798

0
10

10

1123

5347

44

724
0

500

74

76

74

0
4781226

4781226

1728

1042

37

134
0

134

185

15

19

11

552

5685

130

945

105

0
4716

3661

77

32

47

647

252

662

252

0
23

0
23

23

0
0

0
0

0
0

0
0

0

0
0

0
0

0

0
0

0
0

0

0
89

0
0

0
0

0
0

0

0
89

0
89

89
0

89

0

0
0

0

0
346

346
0

94
0

94
0

94
0

94

0

0
252

0
0

0

0
252

0
252

104

102

46

211
0

0
211

211
0

211
0

211
0

211

0
1822

0
1732

0
1732

0
1732

0
1732

1732

0
90

90
0

0
90

90
0

90

0
1800802873

0
177348

0
177348

0
177348

0
177348

9403
0

9403

26727
0

26727

14573
0

14573

8700
0

8700

0
1986

1986

4715
0

133

4582

0
503

503

0
110741

110741

0
73735

812
0

0
812

812

1720
0

1720
0

0
1720

1720
0

243

48

1351

78

20940
0

0
20940

0
5981

5981
0

1625

4356

0
14959

9056
0

9056

0
4837

3097

1740

0
1066

1066

50263
0

0
24040

24040
0

0
24040

24040

26223
0

26223
0

18289
0

18289

0
836

836

0
824

824

0
6274

44

6230

0
15653

15653
0

15653

0
45121

0
45121

0
45121

0
45121

45121
0

45121

0
5383

5383
0

5383

0
1319812

0
1310582

0
177388

0
177388

0
168321

36564

8792

1349

114648

6968

9067
0

4797

179

199

2275

1587

30

874411
0

874411
0

0
29746

29746

0
81449

578

346

54622

760

929

60

7001

1693

22

19

568

1528

424

430

4851

310

40

241

929

2113

3746

81

56

102

534789
0

115

1231

43

707

103

15173

64173

1278

8635

6153

5886

860

602

6742

1370

82

596

542

1897

12

18604

17

65

184

65849

25035

131

188

2104

4606

2972

2134

89320

1352

1689

2457

79026

4293

27096

8436

702

17705

186

289

3486

45943

2325

5853

6542

228427
0

89296

66036

73095

258783
0

216367
0

0
216367

2108

578

1767

5383

2517

4960

3174

7145

19011

1441

13808

2980

7780

35750

3793

660

5135

333

39280

733

76

315

56449

1191

42416
0

0
13347

37

20

4012

5866

19

1561

1832

0
29069

356

19802

1722

7189

9230
0

9230

0
29646

29646
0

29646
0

0
29646

0
5586

5586

4752
0

4752

0
3233

3233

2536
0

2536

13539
0

13539

0
117906

0
6826

6826
0

0
6826

0
6826

6826

1114
0

1114
0

0
1114

1114
0

1114

8880
0

0
6638

6638
0

6638
0

6638

2242
0

2242
0

0
2242

2242

3650
0

3650
0

0
3650

3650
0

3650

30119
0

30119
0

30119
0

0
3252

3252

0
8714

8714

18153
0

14832

3321

0
12520

0
12520

0
12520

12520
0

12520

0
2032

2032

0
37756

0
29227

0
29227

29227
0

29227

8529
0

8057

472

0
15009

0
15009

0
12881

0
12881

124

12757

0
2128

0
2128

940

1188

130716654
0

130716654
0

0
130716654

0
64126067

1125755
0

1125755

0
1642110

1642110

2105210
0

2105210

58824527
0

6835933

4469847

1671272

1451945

1101828

38214881

1685840

1774083

1618898

0
428465

428465

66590587
0

0
42919

42919

0
66547668

96625

89163

62659

3941597

5905756

600131

30910529

680183

23404290

56576

33381

766778

8221
0

6210
0

0
6210

0
6210

0
6210

6210

0
2011

0
2011

2011
0

0
2011

2011

434630
0

1303
0

0
236

236
0

64

172

1067
0

1067
0

1067

0
593

0
593

593
0

593

43943
0

31982
0

104
0

104

0
17874

17427

447

5226
0

5226

3967
0

3967

20
0

20

4791
0

4791

448
0

0
448

398

50

6197
0

6197
0

6197

5316
0

1673
0

1673

2470
0

1783

281

406

1173
0

1173

0
47610

0
12087

12087

2195
0

1764
0

1543

221

0
431

431

80
0

80
0

80

1856
0

1856
0

1856

392
0

0
392

392

0
31000

0
82

82

0
1492

1492

0
4995

4995

0
4476

4476

19955
0

19955

47766
0

0
47766

47766
0

47766

507

0
1327

1327
0

1327
0

0
1327

559

768

0
122513

0
102

102
0

102

0
223

223
0

223

0
93

93
0

93

0
24209

0
859

859

22514
0

353

609

1295

1421

4478

388

6438

38

4445

303

96

129

20

89

70

488

262

111

42

118

31

85

35

388

782

0
166

121

45

0
670

670

0
68565

0
68565

579

1605

63039

2171

1171

0
2995

2995
0

21

2974

0
21149

21149
0

21149

342
0

0
342

342

4835
0

4835
0

587

4248

163

168905
0

0
304

304
0

304

0
82936

309
0

309

0
366

366

32003
0

11217

13884

6902

0
50258

18836

1026

119

114

3259

134

452

225

3277

62

2627

520

204

131

18651

163

93

21

344

0
62508

62508
0

4449

234

1134

344

55357

272

194

524

12093
0

0
1072

1072

667
0

667

0
10103

6074

421

3608

0
231

231

20
0

20

517
0

517
0

517

0
3710

0
2492

25

2467

1218
0

1218

0
6837

6837
0

6328

509

70

0
41865

41865
0

41865
0

41865
0

4267
0

4267

0
437

55

382

0
10452

279

7362

2029

782

26049
0

61

22507

3481

0
660

179

135

346

311083
0

0
2618

0
2618

2618
0

0
2618

2618

17298
0

0
1633

1633
0

1633
0

1633

0
10019

0
10019

6953
0

6953

0
3066

3066

0
5646

0
2104

0
1202

1202

0
902

46

856

3542
0

0
3542

3542

0
291167

61628
0

0
61628

53
0

53

322
0

322

60198
0

60198

0
168

168

887
0

887

0
97844

0
49744

32
0

32

0
40846

593

40253

0
160

160

0
5005

5005

0
377

377

0
58

58

0
2991

2991

0
52

52

0
223

223

19223
0

190
0

190

0
18582

18582

257
0

107

150

0
119

119

75
0

75

28877
0

0
28877

28877

12290
0

12290
0

206
0

206

0
4934

4934

0
627

627

429
0

429

6094
0

6094

0
119405

0
119405

1047
0

1047

1630
0

1630

0
774

506

268

384
0

384

0
415

415

0
1507

1507

88
0

88

1315
0

1315

109479
0

108928

69

285

125

72

2579
0

2579

187
0

187

0
5599

0
5599

0
5599

0
5599

5599

90162
0

90162
0

0
81200

0
81200

81200
0

519

96

103

3713

76109

22

17

43

12

566

0
8962

0
6464

5519
0

5519

150
0

136

14

0
795

795

1704
0

1704
0

1704

794
0

0
794

794

0
4615380

0
4615380

0
13179

13179
0

0
10821

173

6549

70

435

646

1473

14

1461

0
2358

2358

0
4594794

33054
0

0
9664

7242

34

172

2216

0
23390

2707

7401

501

11666

1115

4561740
0

0
32579

7515

4153

20911

11834
0

1395

10439

0
4238

4238

4494642
0

27139

68002

10000

29036

466343

27083

36958

43308

116

30706

290959

16200

68455

70989

175411

140988

61463

431078

2500408

6729
0

6729

0
7558

7558

0
4160

4160

7407
0

7407
0

7407
0

465

1170

540

979

4253

5162
0

0
5162

5162
0

5162
0

5162
0

5162

11183
0

0
11183

11183

0
55677

55677
0

10313
0

0
5028

0
1720

799

921

0
3308

333

231

511

2233

5285
0

0
89

43

46

0
5196

130

5066

24215
0

0
24215

0
2968

2968

0
10542

10542

0
10705

10705

5642
0

0
5642

0
5642

5642

15507
0

0
15507

4613
0

210

4403

0
10894

2239

8655

130885142
0

3447
0

0
3447

0
3447

3447
0

231

3216

0
92209

1210
0

1210
0

0
1210

1210

0
352

0
352

0
352

352

90647
0

0
90647

0
90647

90647

0
1844989

30854
0

30854
0

0
2762

2762

1064
0

1064

0
1836

1836

1791
0

1791

4455
0

4455

12029
0

12029

0
1716

1716

0
5201

5201

1814135
0

0
1807143

0
1669894

1669894

0
2662

420

2242

0
134587

108504

18777

753

1518

2675

2360

0
6992

0
4128

4128

2864
0

2864

7873
0

0
7873

2757
0

2757
0

2757

4597
0

4597
0

4597

0
519

0
519

519

128911627
0

0
388863

0
7098

0
504

504

0
6555

6555

0
39

39

377113
0

0
207013

202534

4479

32876
0

32876

137224
0

137224

0
4652

4368
0

15

198

3836

319

284
0

284

12995
0

12995
0

0
12995

1839

5418

2803

187

2748

0
6399250

0
35564

35564
0

20637

11856

1908

1163

710
0

0
710

639

71

0
2674662

0
118224

2576

10

3268

73

955

78

11

23

17

1213

867

142

29

104763

123

105

166

343

2337

351

518

119

15

23

35

44

20

0
2550613

5267

4749

1013

15

41851

59

4737

3847

73

1996518

23

124

128

153

28039

6854

9866

1735

7903

1134

315

436187

23

0
4110

905

3162

43

42
0

42

1620
0

1620

0
53

53

0
3719

0
3719

3719

2623
0

2623
0

2623

1008
0

1008

120943
0

34813
0

12

3155

39

33

639

12

1520

302

70

301

15569

54

3137

4624

4579

665

40

62

0
86130

50

112

84546

48

208

497

496

17

82

74

37817
0

0
37817

58

48

38

12275

2364

16

22844

174

0
3522204

0
3522204

647

214

4911

7659

788

31473

14

679

23179

617

37127

617

176028

21363

26

112322

2230

13927

165

84

4385

1343

6063

1779

1803701

24

634

166

532

5244

411

303

931

14066

9158

339

614135

537

2789

1620

157

15

61

11018

1382

10813

4176

5415

1013

18514

20401

2328

1405

268

61562

4680

17813

259

1076

1434

166

482

7765

123

1103

5618

4857

4891

379112

1496

1693

88

193

1102

3090

25445

3730

687

66

4594

174

4634

557

4046

363

109

874569
0

0
874569

0
3604

3604

19
0

19

0
44

44

0
734

734

155697
0

15757

237

527

139176

530063
0

373853

3952

58302

23316

14607

33791

22242

16928
0

224

13

16546

82

63

0
1873

43

1830

178
0

178

0
1652

1652

0
157965

147281

443

5834

1454

2953

5506
0

5506

306
0

306

1145122
0

1145122
0

1144396
0

1666

637805

1791

40

4266

3689

17

20

180

78

2526

7307

578

1557

483

40

11

18

1631

13

50

161

22543

22

861

168

66

316

334

151

50

1421

174

13

356

17

106

604

1062

115

147

16

13

2943

1115

154

56

2020

24

14

106

462

67

97

2441

35

648

321071

18

1822

34

647

68

57074

4273

959

209

412

35

89

238

138

2450

3648

1509

2737

22

7773

236

13

15010

13

12

50

3228

2203

4035

2721

191

14

73

936

314

7447

10

534
0

140

394

192
0

13

45

52

12

70

3670
0

0
3670

3670
0

3670

0
442

442
0

0
442

442

41371846
0

41371846
0

6753287
0

250994

67472

1491009

329920

1180372

16869

26727

365668

110017

9251

19321

149483

361535

36372

637936

317927

2733

32499

25744

12425

6207

559967

54393

9781

8165

18411

391356

260733

101418
0

101418

0
124726

3501

17248

19956

84021

7434
0

7434

0
33356744

9395029

144600

23151596

545508

120011

0
21181

21181

602041
0

602041

0
292269

32397

143843

20860

73673

21496

0
68153

2310

5493

60350

0
11284

11284

0
20538

20538

0
12771

12771

0
7121

7121
0

6279
0

406

77

880

3467

261

1188

842
0

67

451

324

0
743300

0
743300

33372
0

33372

17030
0

2669

14361

0
674214

274

206500

14558

2279

219

37539

268

22450

3708

9457

19079

84

28

338099

36

11246

3804

2948

16

807

815

18684
0

18684

6700
0

0
6700

0
6700

6700

0
668624

43341
0

43341
0

43308

33

0
71806

0
420

420

66
0

66

0
85

85

1272
0

1272

4042
0

4042

0
2406

2406

0
63515

954

753

45

10551

89

2849

38

19

236

43

195

4959

1595

5446

2337

93

22306

471

1334

1195

8007

0
1993

0
1993

1993

551484
0

0
103040

19561

58898

24581

3610
0

3610

3047
0

3047

0
290947

290947

0
826

826

75160
0

28099

9984

15558

21519

0
15317

9898

3104

2315

0
17033

17033

0
8224

235

1435

302

922

5330

0
2763

2763

31517
0

237

1393

113

5122

14605

10047

0
1273

1273
0

1273
0

1273

936
0

0
936

936
0

936

0
4385

0
4385

0
4385

4385

0
349

0
349

0
349

349

0
485

485
0

485
0

296

189

21835
0

0
21835

0
2425

2425

12554
0

64

39

22

4316

8095

18

0
480

480

5486
0

4819

667

0
890

218

672

0
77257698

384
0

384

27943
0

0
10557

10557

124
0

28

96

11777
0

766

9467

1544

118
0

118

5367
0

5367

3233
0

0
3233

2975

258

0
83327

476
0

476

332
0

332

59488
0

59488

0
21326

6943

30

3133

4059

2623

392

4108

38

1705
0

1705

0
41369

0
23005

13813

181

4538

2626

1847

0
3676

3676

0
14688

12106

2582

0
76079148

0
347561

347561

8393
0

8393

10234
0

124

62

9763

24

261

0
2618

2618

6839140
0

25268

10845

6752811

128

222

1559

16

5766

29

110

18

38

2804

672

30693

21

39

7472

629

0
19479

6540

68

3131

18

9113

365

244

6535
0

6535

35598
0

35598

152970
0

39

95

2468

15324

38

665

11756

15634

106834

117

0
86259

86259

0
68469831

54049664

12519

220557

38692

140600

7143656

54958

6809185

7188
0

7188

0
91577

1083

90165

329

1765
0

1765

48728
0

2009
0

2009

0
45985

45985

0
734

734

4146
0

1520
0

235

38

646

601

2626
0

31

91

1939

61

504

0
284027

0
284027

284027

0
161270

0
213

87

126

159322
0

158908

414

359
0

359

1376
0

1376

0
16283

0
16283

272

16011

5334
0

0
5334

96

2139

3099

0
43837

43837
0

43837

8606
0

8335
0

8335

271
0

271

11232
0

11232
0

11232

8021
0

5276
0

789

4487

0
2035

2035

710
0

710

0
337147

0
163

119

44

0
66479

66479

0
87

87

0
104

32

72

0
783

783

23306
0

359

22947

0
1976

51

52

54

18

1745

56

0
48

48

0
3407

3276

24

107

0
30280

2905

24175

378

2131

547

86

58

0
252

252

588
0

437

75

76

13
0

13

5034
0

5034

0
180015

307

28

160

11621

206

87

45

3365

20

995

44

604

13419

24

21

180

16

1672

25

141387

22

1164

4603

0
6264

1130

658

22

603

3851

0
789

743

46

7261
0

4515

1136

1236

342

32

0
13

13

0
211

139

46

26

1121
0

1121

147
0

147

2253
0

1577

635

41

6553
0

6553

0
93663

0
93663

15545

40850

86

13279

2025

4884

508

174

1643

13442

68

1106

53

2164
0

0
2164

0
821

821

0
515

515

0
828

828

0
24997

24997
0

0
24748

24748
0

16044

1435

3546

1101

2622

0
249

249
0

249

6017
0

6017
0

6017
0

6017
0

0
6017

6017

0
64282

0
40098

7077
0

0
832

0
832

832

0
6245

6245
0

6245

33021
0

0
33021

0
1013

107

173

733

0
18542

18542

0
4090

51

4039

0
182

62

120

0
7721

169

7454

98

0
1473

1473

0
10428

0
10428

10428

0
3083

0
3083

0
3083

0
3083

3083

10673
0

0
10673

0
10673

10673

8309
0

0
8309

8309

2776
0

2776
0

0
2776

0
2776

0
2776

2776

13673
0

0
1670

0
1670

0
1670

1670
0

1670

12003
0

12003
0

0
12003

0
7475

4550

132

2793

0
4528

519

4009

511213997
0

419402
0

12378
0

0
4312

0
2929

2929

1383
0

1383

0
8066

4700
0

4700

2886
0

2886

0
480

480

30556
0

4769
0

4769
0

4769

0
7370

7370
0

7370

256
0

256

6351
0

0
6351

6351

7879
0

1137
0

1137

3996
0

3996

2746
0

2746

549
0

0
181

181

368
0

368

0
581

169
0

169

412
0

412

0
2801

0
2801

2801

0
5658

5658
0

0
5658

5658

3437
0

3437
0

0
1397

1397

0
2040

2040

2073
0

0
2073

2073

0
2446

0
2446

2446
0

2446

165115
0

69617
0

45347
0

3316

322

41709

17042
0

16968

74

0
3387

3387

0
631

631

3210
0

3210

95498
0

0
20049

20049

27350
0

27188

135

27

0
6801

3252

3549

3073
0

1537

1536

16967
0

957

16010

0
20426

20426

0
832

832

28574
0

4069
0

2812
0

2812

1257
0

321

936

0
23106

9810
0

9810

0
13296

1886

101

412

1195

3347

1382

719

1011

252

2991

1399
0

1399
0

659

236

504

169165
0

27993
0

0
27993

1842

26151

110273
0

0
10445

187

6317

494

28

656

2763

6994
0

6994

0
82996

451

15892

34616

700

216

24282

819

554

43

60

4427

936

5125
0

5125

4182
0

3435

747

531
0

531

0
30899

0
30899

30899

0
213652139

185745004
0

0
185546086

1744794
0

100024

1644770

0
177272813

16739858

3833251

9398581

262918

4204097

27262

911320

49167

4351261

171838

605663

3901152

1942889

66416

828600

49612

347896

120377450

38796

116964

46105

50246

163480

218703

8473007

31492

64789

0
161079

161079

4750636
0

3390531

1166170

193935

0
3046

3046

0
1465405

1465405

0
46720

46720

0
1174

1174

24977
0

24977

0
75442

4279

71163

0
198918

1138
0

1020

118

18396
0

16543

1202

651

0
4487

4487

0
1438

1438

0
6614

6614

0
2987

2987

0
706

204

502

14359
0

13459

900

0
4377

4377

83091
0

83091

61325
0

122

85

6644

546

20184

33744

24693
0

25

127

20410

368

700

3063

0
33492

1874
0

1874
0

1874

11315
0

0
1037

786

251

2735
0

2735

3303
0

263

3040

1056
0

1056

0
3184

3184

17684
0

6206
0

3020

720

1951

515

11478
0

2245

724

13

2726

27

4965

778

2619
0

2619
0

193

159

2267

11782
0

3034

8748

0
27726189

0
35993

35993

0
1678

1678

763
0

763

0
263

263

0
754115

19
0

19

0
4248

36

419

24

3565

25

167

12

517
0

517

12329
0

41

6267

83

5240

698

0
2762

355

2407

47
0

47

1568
0

1568

2059
0

2059

0
12595

12091

504

0
129374

87

109535

5800

9606

788

28

38

3492

1798
0

1798

261
0

261

52
0

52

158
0

16

61

81

0
21651

157

1222

251

148

228

352

150

92

19051

531978
0

76

531531

371

154
0

47

61

46

2954
0

2954

0
893

893

0
5758

5758

0
114

114

0
22826

10945

7405

4005

193

23

222

13

20

71
0

71

33695
0

7080
0

7080

0
26615

7823

1985

16807

0
319022

62738
0

14143

22781

13939

1556

91

81

67

72

9960

48

7927
0

7927

0
1307

28

1279

0
3274

653

2621

0
4745

4745

0
532

532

0
9719

6401

3318

27472
0

26062

114

1015

78

203

0
44277

44277

63370
0

62230

1140

82233
0

82233

0
3321

3321

3492
0

74

50

3368

360
0

360

4255
0

1730

2525

105827
0

0
3507

3507

14976
0

651

833

1128

12229

135

0
738

321

417

1019
0

1019

6181
0

6181

26074
0

657

669

7982

547

457

471

232

14673

112

274

2305
0

2305

0
2690

1603

1087

0
47800

32

46932

13

823

537
0

67

470

26459937
0

0
19236

252

61

127

181

1034

946

16532

41

62

0
1216

1216

6737
0

6737

10968896
0

1303

6827

910

1736

6976

79

4931

294

6759988

2444905

150

34

14

17783

67

575

4014

27

701

587256

4243

112

1572

36

56

5975

1118332

3142
0

1698

1444

158476
0

486

643

9100

10582

14

54

128480

3238

1320

2258

881

22

13

54

19

630

682

1471
0

1471

0
79705

78658

19

31

217

96

649

14

21

0
71985

2414

75

69496

0
57377

898

14726

93

2957

73

4946

17708

565

15322

89

0
15087010

15087010

4163
0

4163

523
0

523

0
62

62

516
0

516

0
3589

3589

0
7209

7085

124

0
574

574

0
376

376

0
2499

2499

104961
0

0
1101

1101
0

805

296

43017
0

1936
0

14

165

1757

41081
0

2510

733

2410

5950

434

13802

15242

2617
0

29
0

29

0
207

207

1084
0

1084

0
1297

1297

14654
0

712
0

712

10217
0

10217

0
3725

1472

1082

1171

0
5324

0
1535

1535

0
1276

1276

0
105

105

2408
0

864

1544

0
25098

0
652

652

9879
0

6965

2914

0
3152

2131

1021

10596
0

86

311

165

10034

0
819

819

13150
0

2207
0

2207

10943
0

10943

1722
0

1722

0
4296

4296
0

4296

21999
0

0
21999

21999
0

21999
0

21999

0
55455

3238
0

0
3238

0
3238

3238

0
14762

13618
0

13618
0

8049

5569

0
1144

1144
0

1144

37455
0

0
37455

37455
0

1326

2152

32894

1083

0
34018087

33998893
0

15577
0

13797
0

155

162

5685

231

2879

652

781

127

68

108

123

2826

1780
0

1780

2139
0

0
1369

1369

0
770

556

116

98

893
0

0
893

893

33709761
0

0
34082

231

5985

13

434

25954

1465

0
10333

2645

369

126

157

186

159

419

5056

1216

3001
0

283

1144

162

527

197

128

296

95

169

0
33644156

2087

984

1324

10510

22417

1294105

82084

108460

5576

451872

3675

5994

4667

54161

51252

1344

7600

5671

1485477

92924

695

590

4205

46587

161107

239418

30268

39321

773312

9773

10911000

17023954

47707

206116

82455

69

4323

213123

117475

20175

4631

15668

0
871

871

909
0

451

458

1204
0

1092

112

0
15205

117

72

88

5755

588

6825

818

93

849

270523
0

270415
0

617

173

815

8538

146

174

569

7632

65

664

228

161

76

25251

178675

1886

15

43201

1529

0
108

108

19194
0

11368
0

0
380

380

0
10988

298

3631

7059

0
7826

1846
0

770

1076

2347
0

2347

0
3633

3633

261951773
0

179394
0

0
35890

1461
0

1461

0
34429

777

543

33109

59079
0

0
16404

16404

13313
0

13313

6112
0

2674

3438

933
0

933

0
6884

6461

161

262

11044
0

1010

1391

8643

4389
0

66

1563

1734

1026

84425
0

0
84004

230

66

621

147

5206

23

62

50548

700

19467

261

122

106

6445

0
421

239

125

57

88949
0

0
12435

12435
0

523

11912

5858
0

5858
0

5858

18283
0

853
0

853

0
200

90

110

11352
0

5311

6017

24

0
113

113

5149
0

3388

1761

0
616

139

477

0
45217

7088
0

6574

192

322

6276
0

6276

0
9372

172

4450

4467

283

0
1219

1219

0
11595

11595

0
3918

3918

1887
0

1887

731
0

731

44
0

44

1967
0

1967

1120
0

1120

1264
0

0
1264

803

461

2650
0

2650
0

2650

1282
0

0
1282

1282

0
1960

974
0

974

986
0

986

0
2599

2599

1865

13325
0

0
9137

0
2972

2972

6165
0

6165

0
4188

4188
0

4188

29830
0

29830

0
247

247

0
832

832

296
0

80

216

1312
0

1312

0
1795

0
1795

0
1795

1795

0
1722411

1186285
0

0
3115

3115

13815
0

25

13790

0
1166316

980

1829

550

296

7560

548

26

626

8922

968749

131

1420

128

32

13

1973

496

10

1701

1803

2363

247

131

4015

10

1689

864

1732

232

108

1634

18981

12

1820

46

1800

148

899

76

12

415

34

576

283

65

67

19742

2226

145

1484

10

141

10

17

773

24

20

2794

13

22

408

65

3314

12

28

878

4276

456

25

71

35

941

2847

636

1772

75

2946

26892

15

53

30746

26314

38

1353
0

575

778

0
1686

1686

0
536126

117
0

117

0
363249

1387

5049

442

543

179

610

362

163

46

4416

2852

1245

104

17

7067

151

1774

344

2396

212

1187

307

78

15

16

1745

4191

61

2862

281610

114

182

2194

513

147

26

149

244

219

12851

549

1031

296

1285

91

1303

20281

36

11

41

255

0
16537

212

1663

171

88

4705

758

1639

5717

767

575

242

0
156223

2065

43578

1687

2162

567

77693

28471

1413
0

811

602

25937

0
230449

0
23528

0
2541

2541

0
3461

3461

5251
0

5251

0
1485

20

1465

8908
0

8908

0
1644

920

724

238
0

218

20

206921
0

0
93934

60

13

5346

672

1025

16

86765

37

0
336

336

15868
0

566

20

93

1282

2853

226

12

10703

88

25

69389
0

108

232

49675

4799

2901

96

373

5490

48

3063

19

2183

279

123

1126
0

1126

0
6709

410

6299

0
996

671

100

63

162

803
0

115

675

13

17760
0

40

16673

1047

16771
0

16771
0

876
0

264

324

32

216

40

0
1593

1593

0
3826

56

3770

2810
0

2182

628

0
6191

6191

0
923

923

0
552

552

0
2996111

2996111
0

0
3104

3104

0
300

300

115447
0

95707

1266

3209

15265

0
4285

2302

956

1027

0
2125

2125

61889
0

6838

55051

0
2808961

152

12

414

11260

65

1014

21661

280

2596

281

3447

9411

20568

247

1680

410

18719

17

14205

317

15445

1074321

7040

2711

38

722

3378

281

2396

100

451

824

1276

49

878

382

201

156

880

3963

3000

160

3733

425

2182

4551

51856

64

964396

123626

630

31074

2293

324

356116

41

36262

5493

487

5519
0

5519
0

5519
0

5519

0
60812354

0
48576042

0
94

94

0
338

139

120

38

41

12404
0

1931

1077

2167

2809

4420

0
2914

2914

131

685
0

685

303

6292
0

6292

1484
0

1484

0
60

60

0
481

291

190

946
0

946

8075
0

8075

178939
0

178939

1917

156

31098
0

31098

0
20032736

550

10025

2202

20019890

69

75

0
2195

491

1704

9215
0

27

8487

58

305

10

328

498

568
0

108

150

310

596

0
5954

5954

0
53470

180

14601

54

176

9837

57

959

19967

294

547

59

56

19

2289

4375

16579
0

16579

0
279152

20597

21

125484

3032

38009

11824

31

62

27

76

25

70307

21

71

7451

303

14

1721

76

0
46152

46152

0
25369778

445018

32289

19700722

5191749

0
266

210

56

10
0

10

3535

8732

0
27789

13

46

16

13991

13723

3362

0
24201

1263

464

2201

12040

7706

527

61

135
0

135

1188

93

151

51
0

51

0
351

60

291

136

461

121

1056

314

10496

94

0
2389389

24156

668

1413

20504

93

48320

894

114

14189

2247037

31960

41

0
15052

6040

9012

80

15454

5536
0

5536

0
4171

50

2948

1173

472

12632
0

5213
0

5213

0
5020

5020

430
0

430

0
1969

1969

11096413
0

0
60

60

0
109

109

60
0

60

8712
0

4576

4136

4489
0

4489

11
0

11

136
0

136

55248
0

1142

199

12954

9416

9502

69

4271

5796

25

923

11

318

8110

1017

64

1431

11027588
0

2623

49

148

28073

132

10916431

59698

532

302

3171

1204

13365

840

1020

75444
0

75444
0

12361

58958

4125

706164
0

0
481646

8811

462396

2527

1279

5964

669

0
7267

351

5127

1789

0
40007

5358

4926

24700

5023

0
115273

3737

59000

278

51185

812

261

55660
0

1614

437

3737

4719

24469

19402

1282

0
6311

6311

0
112029

0
107179

1736

311

85388

19656

88

0
4454

4454

396
0

106

290

43632
0

0
32309

17605

37

95

50

1651

12255

616

0
41

14

27

0
7474

3419

91

23

79

1229

465

155

333

1680

0
3808

296

39

3473

187450
0

307
0

61

246

4740
0

3811

444

485

1385
0

1385

0
85525

85525

0
38316

63

21470

107

189

14768

922

553

174

70

57177
0

3978

5854

40

36

3979

541

1424

100

7243

1687

225

618

1936

2272

9200

18011

33

2548
0

2548

144255
0

19028
0

0
11225

4812

668

5745

2974
0

2974

4829
0

4256

573

0
125227

0
117

117

124779
0

21795

2157

38

5426

609

86574

167

2842

196

747

170

287

168

69

225

156

383

513

944

1313

0
331

331

2126

0
833179

14073
0

0
14073

14073

0
819106

0
12529

12529

3121
0

3121

0
338

338

0
2848

2848

3453
0

384

3069

796817
0

6854

18747

3216

692765

243

2064

32

48747

13604

523

10022

0
62661

0
62661

15179
0

15179

0
33693

33693

13789
0

13789

6143
0

320

274

5549

994

0
2966795

2966795
0

150167
0

150167

0
2816628

2816628

496
0

496

0
190270937

190270937
0

0
2987208

539

121374

49604

2815691

0
135966026

5519158

8904757

19369750

298562

1425846

258063

96649348

3540542

6714115
0

282274

80421

26386

142124

8788

166354

65077

5942691

0
312434

312434

0
42916

42916

11203412
0

3439898

4375779

1123713

2264022

0
62731

62731

0
19852892

19852892

0
45033

45033

76782
0

76782

0
108630

37383

71247

0
203920

57084

146836

49919
0

22570

27349

10631079
0

63995

80664

3646482

385334

75615

4335096

90998

404230

1548665

0
503375

503375

0
1510465

1466547

43918

827522
0

12661
0

12661
0

537

781

10658

347

338

36568
0

25175
0

2950

743

20677

805

0
10337

1712

233

253

980

1018

5602

334

205

1056
0

1056

7225
0

0
7225

1102

6123

0
143307

364
0

364

2051
0

164

145

1474

268

0
106180

193

368

39563

5639

8997

94

1457

40

35719

62

13934

114

18087
0

492

158

17

168

2558

17

1092

16

163

13260

114

32

1385
0

1385

5053
0

2333

2720

0
1972

1108

864

868
0

868

3063
0

3063

4284
0

2213

86

1931

54

0
4492

4492
0

539

3953

189725
0

0
187938

868

112

77

65

34

867

2811

83

31

1942

115280

1890

4060

407

326

1605

1865

1360

22

12

13

11932

160

201

289

138

118

58

216

89

116

1941

35

144

15

152

171

93

503

11

372

99

11102

26253

0
1787

593

1194

6173
0

0
6173

741

5432

427371
0

0
197415

195202

2213

229956
0

1070

249

2199

1915

501

4230

4485

11817

3013

15

319

278

29196

1958

777

902

383

7714

72

686

1252

402

117

132

258

1139

4914

4382

6090

10842

5533

113533

866

646

7749

140

182

51417
0

0
1552

0
1552

622

17

50

81

353

373

56

0
20286

1092
0

1092

12120
0

436

11684

7074
0

7074

28486
0

11309
0

11309

48
0

48

6649
0

5172

768

709

0
10219

2031

3414

929

94

3751

261
0

261

0
1093

548
0

548

129
0

42

17

70

0
416

416

0
574739

886
0

467
0

426

41

419
0

419

0
36751

0
783

363

233

187

5049
0

5049

1047
0

1047

0
4863

2730

1715

418

2834
0

2834

1004
0

1004

0
1407

1407

19764
0

384

1757

342

1057

25

8633

6365

1201

2001
0

0
2001

2001

0
1518

1518
0

1518

6184
0

6184
0

2580

313

1942

1349

26674
0

26674
0

26674

0
6643

3301
0

3301

0
2903

2903

0
439

439

91796
0

0
1778

1778

0
10432

10432

3006
0

3006

578
0

578

367
0

367

54
0

54

0
14888

14649

239

18572
0

18572

37433
0

29

80

272

20

1699

833

4124

196

20601

11

30

22

787

914

744

3966

244

1105

187

1569

4688
0

4688

402286
0

401649
0

389533

7393

4723

637
0

637

66841
0

66841
0

0
36522

36522

0
4132

4132

0
26187

26187

12259
0

2836

7125

2298

3254
0

0
3254

3254
0

0
3254

2890

245

119

0
2789

2789
0

2789
0

0
2789

1680

1109

1089099
0

0
12424

12424
0

0
2165

2165

0
10259

10259

0
153970

0
105078

0
119

119

0
3433

29

40

3364

52738
0

52682

56

0
872

872

15890
0

15890

7631
0

2531

5100

0
750

261

489

0
400

124

276

28
0

28

0
1065

43

1022

0
15631

15631

0
1586

1586

0
4678

4678

257
0

257

0
47235

39
0

39

4065
0

4065

0
1695

1695

2409
0

1174

1235

0
865

865

0
266

266

0
10521

10521

0
20

20

19705
0

10511

99

132

1450

18

17

49

7429

3938
0

1478

2460

0
667

667

460
0

460

0
11

11

582
0

54

528

0
1694

1694

0
298

298

0
1657

1657
0

1657

0
4771

0
4771

4771
0

4771

432383
0

0
4872

0
113

113

1479
0

245

35

139

307

441

94

26

82

110

0
1236

1059

177

0
31

31

15
0

15

1998
0

1998

0
90499

4175
0

38

19

26

28

371

3196

38

21

49

73

12

28

14

166

96

96
0

96

0
63

63

57
0

57

43
0

43

0
28

28

0
71

56

15

197
0

76

121

0
7538

27

97

229

55

7105

25

0
78231

53580

898

2133

12310

9310

0
1247

16
0

16

0
1184

1184

47
0

47

0
4071

0
1072

1072

48
0

48

2641
0

2641

0
62

62

248
0

248

23
0

23
0

23

15685
0

0
4781

4781

88
0

67

21

0
2216

2186

30

0
26

26

223
0

223

2417
0

2417

0
197

197

0
38

38

236
0

236

5463
0

345

145

11

3113

1569

227

53

8967
0

8967
0

513

430

73

29

246

578

196

242

742

16

4882

14

891

115

278
0

278
0

278

11
0

0
11

11

89
0

89

1363
0

85
0

85

0
1278

1278

14
0

14

345
0

345

495
0

51
0

51

103
0

103

0
135

135

206
0

45

98

63

67790
0

0
1189

339

30

820

1201
0

1201

3949
0

3949

16
0

16

44
0

44

0
60

60

0
60084

59967

117

1247
0

1247

1973
0

1573
0

1573

0
400

39

361

0
46325

46325

170
0

0
17

17

17
0

17

136
0

136

0
8729

0
7347

16

148

92

437

234

71

116

6082

110

41

185
0

142

43

1197
0

449

100

648

0
179437

0
23237

23237

0
156200

729

654

91

1061

4997

39289

109379

4182
0

0
4182

4182
0

4182

0
46443

601
0

601
0

601

44653
0

0
5663

5663

23876

0
15114

5855

9259

0
1189

0
1189

1189

6747
0

6747

0
1036

1036

138181
0

0
32074

423
0

309

101

13

325
0

325

2858
0

931

1734

10

183

28468
0

70

499

68

120

10024

15849

71

1310

457

8764
0

0
4979

4979

3785
0

3785

5235

86528
0

66941
0

66941

12083
0

1996

5985

1812

318

260

1602

26

31

16

27

10

0
3332

3332

0
4172

4172

5580
0

0
5580

5580

14230
0

14230
0

221
0

221

0
5222

3233

1853

57

61

18

5536
0

23

451

79

4983

983
0

983

2268
0

105

2163

0
13663

0
13663

0
1674

1674

0
11989

11989

0
11218

0
11218

6166
0

1111

3534

1521

0
5052

5052

0
814

814
0

0
814

814

761
0

550

89

122

6038
0

0
5462

5462
0

152

205

1285

244

60

3352

164

0
576

576

105093
0

105093
0

0
16359

480

512

8348

354

514

33

311

956

85

3440

1258

68

0
2235

2235

0
329

329

0
71

71

955
0

955

0
445

445

0
209

68

96

45

0
1144

1083

34

27

0
23

23

12
0

12

0
329

329

0
3856

3856

0
3368

3261

107

0
131

131

0
114

114

0
23086

23014

72

24370
0

71

24299

0
30

30

38
0

38

0
235

235

0
333

249

84

0
12

12

0
19

19

89
0

54

35

0
21301

21301

2391
0

2391

0
1339

1339

109
0

109

0
13

13

115
0

115

0
186

93

93

0
76

48

28

0
136

136

0
1635

1635

0
137145

7362
0

7362
0

579

6783

0
128176

42
0

42

0
17

17

370
0

370

0
52920

14

44

91

30

25

43

341

22

20

513

7764

45

10

42959

932

41

12

14

2868
0

43

926

385

1451

63

138
0

84

37

17

0
279

279

6305
0

6305

3753
0

3753

61484
0

3121

29

58252

82

1607
0

52
0

52

145
0

145

0
301

108

193

310
0

310

530
0

28

448

54

0
32

32

237
0

237

776
0

0
776

0
776

0
776

776
0

707

69

628124
0

623889
0

0
623889

623889
0

623889
0

621296

2593

4235
0

0
4235

0
4235

0
4235

4235

0
156093

0
156093

0
156093

0
156093

12415
0

3829

149

8437

0
143678

143678

18425
0

0
18425

18425
0

13614
0

13614
0

13614

0
4811

0
4811

4811

2940105
0

314010
0

314010

0
2626095

2626095
0

2626095
0

0
2626095

2626095

182080920
0

233930
0

233930
0

0
233930

55357
0

6910

184

17695

2728

17590

2128

8122

0
4104

600

3504

2054
0

1414

640

0
704

704

1026
0

1026

2617
0

2617

2414
0

2414

0
10573

10573

22531
0

22531

0
291

291

0
39274

39274

92985
0

1390

11339

30741

49515

0
2172292

2172292
0

30780
0

1937
0

1937

28843
0

28843

50383
0

50383
0

50383

93914
0

0
22276

21216

1060

16286
0

7073

1857

5381

647

1328

3542
0

3542

28198
0

11932

2835

13431

0
23612

23612

647849
0

0
647849

647849

0
54440

0
54440

54440

0
34521

0
26396

11254

14226

916

8125
0

8125

281656
0

0
2503

2003

500

95306
0

1917

1094

950

3987

3406

83952

0
166035

437

988

55

70

337

304

1305

12

412

157

460

627

142472

764

426

52

442

285

8512

4343

3064

511

0
3366

404

1841

1121

14446
0

14446

0
12741

12741
0

6886

5855

966008
0

0
282839

29429

2661

663

7597

3242

674

26503

202274

331

7878

1587

0
89412

36242

13363

39807

0
5043

5043

0
4295

4295

0
482163

482163

0
30704

30704

0
35210

22759

12451

0
24088

24088

0
12254

7992

4262

121567683
0

0
121142575

0
1446775

352931
0

352931

0
1030280

931812

98468

63564
0

63564

0
113726

113726
0

113726

12168509
0

0
2683382

1821583

861799

0
9485127

2598230

6886897

1308413
0

734942
0

544912

190030

496232
0

496232

0
77239

77239

34408
0

0
34408

34408

11623388
0

0
11623388

4340736

1302288

5392582

587782

3247015
0

347455

1071864

1493583

334113

0
359665

359665
0

4479

15019

4759

1720

9869

32911

186092

104816

1135472
0

1016403
0

1016403

119069
0

32028

87041

0
1245410

0
1245410

1245410

83671637
0

0
856281

856281

76704576
0

5804113

603873

2059152

20226028

1619864

3215220

28987943

8022154

634101

985942

1122560

1915510

1508116

0
6110780

6110780

0
1243231

0
1152247

1152247

90984
0

90984

0
3544926

0
3544926

686167

35420

174216

235980

36273

312761

8353

4173

15941

66793

132577

130277

1671

5085

24532

270057

16734

1161

418766

7936

959153

900

425108
0

0
34439

0
34439

14545

19894

48810
0

0
48810

48810

341859
0

0
341859

341859

24658
0

0
24658

5058
0

5058
0

5058

19600
0

19600
0

19600

0
15393

0
15393

12192
0

12192

0
3201

3201

0
56839723

56839723
0

8298645
0

620506
0

620506

136545
0

136545

113963
0

69325

12601

6012

5313

5582

15130

1756082
0

1756082

0
547612

547612

0
134871

134871

47359
0

47359

0
167282

20539

43716

103027

0
55076

55076

0
489624

212459

153025

20419

57795

13210

2779

9267

20670

3979520
0

22256

40928

33716

25501

98553

166708

9250

10217

206794

4794

20217

1158965

15546

14262

10463

7668

8546

7718

409069

22904

51843

32586

286555

386936

46779

25401

471501

174508

26552

10390

18950

36715

116729

156556
0

93378

15342

47836

93649
0

18548

75101

0
8453

0
8453

8453

235579
0

235579
0

90

77

5275

162165

163

383

645

66742

39

0
2910

0
2910

2910

6473
0

6473

349266
0

327675
0

327675

21591
0

21591

47838156
0

116585
0

52019

58330

4216

2020

0
28918

283

2458

3753

10463

2316

9645

0
39042

39042

2744308
0

2744308

0
2494

2494

19012
0

19012

0
59311

59311

0
460544

360881

1373

3058

57712

1743

4074

2509

185

2675

1799

1115

3478

4476

634

12421

2411

0
3613

870

2743

4974
0

4974

0
44896

8114

6220

2316

2707

25539

321906
0

317016

4890

7083
0

7083

0
95315

11148

77507

6660

0
30384

30384

0
57773

18487

39286

0
12808

2331

5711

1067

3699

0
77013

1540

5971

18973

6433

10041

34055

0
903001

1255

66266

68415

7782

12486

17141

7797

1300

10724

2407

12401

10196

12044

3670

27716

27482

25724

36630

143736

4630

7319

3500

8906

9738

4601

5370

64568

5105

34238

12186

3489

1057

86771

123607

6175

22054

4515

0
4896

4896

0
3571

3571

0
18272

18272

0
229241

30205

28593

46319

65219

12133

17352

29420

0
15569

15569

4273
0

4273

63583
0

5463

15001

2669

21814

201

4517

13918

0
3786

2218

1568

0
3763

3763

80434
0

2055

78379

30237
0

30237

19193
0

19193

8918
0

8918

2290
0

2290

72228
0

3662

1399

4593

31371

1527

816

7272

16308

5280

13172
0

6203

4147

2822

0
73345

73345

0
63730

3586

4085

5018

27020

6826

3129

14066

0
76921

3536

3036

37432

4961

7912

20044

4448
0

4448

22557
0

22557

2127
0

2127

21839
0

21839

195692
0

195692

2360
0

2360

0
4677

3845

832

76332
0

76332

113342
0

113342

81448
0

1631

2158

19739

57920

113540
0

105033

2022

6485

0
41383392

64716

15640

188026

17328162

1042424

6010090

715010

544019

1776510

4510982

7025405

673511

897590

72988

352839

165480

0
100241

100241

1227241
0

0
1227241

1227241
0

145786
0

2742

10812

14431

5273

2849

729

595

324

703

99937

2701

4490

200

0
116335

2264

12864

724

2280

1081

2412

8321

70438

2622

1382

582

5713

2451

3201

180137
0

161512

18625

0
96144

96144

0
4262

4262

270739
0

270739

413838
0

143224

4003

981

1702

1365

1637

621

2821

193434

9216

976

29600

21806

2452

431

0
12743

12743
0

12743

0
75709

75709
0

0
68343

2636
0

2636

0
8396

7461
0

7461

935
0

852

83

0
57311

276
0

254

22

0
49124

845

48279

0
7767

7767

144
0

16

66

62

0
7366

7366
0

0
3251

3251

0
4115

4115

105416
0

0
105416

53783
0

0
53783

53783
0

100

382

3506

41765

621

244

301

486

97

180

1047

4993

61

0
4509

0
4509

4509
0

4509

0
47124

47124
0

29559
0

98

8730

2462

132

14896

3241

12569
0

298

11659

612

0
611

611

4385
0

4385

0
10690

10690
0

10690
0

0
10690

1054
0

135

770

149

0
603

603

1467
0

1467

0
2566

2566

0
5000

5000

0
834489324

0
1305419

1305419
0

0
23374

23374
0

3822

19552

0
1282045

0
1036

1036

65123
0

16111

7381

1399

6136

12565

14356

7175

0
21533

21533

0
13904

13904

0
190

190

0
12604

12604

1090212
0

1090212

0
58944

52563

6381

18499
0

18499

2880389
0

0
2813635

71242
0

0
14274

14274

0
8985

8985

3546
0

3546

44437
0

44437

2663616
0

2227549
0

2227549

0
39639

39639

289824
0

12032

22644

14102

241046

56962
0

56962

49642
0

15969

33673

78777
0

0
78777

78777

0
45904

45904

0
20850

20850

18811

657507659
0

0
69693526

3438
0

0
3438

3438

0
53044429

5030

34488599

219186

14943863

3387751

0
38443

7712

1479

2137

102

56

398

26434

125

0
157413

24485
0

1012

23473

1435
0

1435

3070
0

3070

120014
0

120014

0
8409

8409

165682
0

20705
0

20705

0
2049

2049

3299
0

3299

23793
0

4900

18893

39658
0

629

578

36

12851

152

25

2146

9110

334

127

1094

12491

85

31023
0

1075

29948

0
6017

3374

2643

19388
0

7927

11461

19050
0

6398

4163

416

5675

1903

495

700
0

700

0
1433639

0
622

622

1357834
0

76

254

64906

74

80

311

110269

221

7531

12055

1811

978

253

328

41

663737

1491

5656

3878

29

2721

14819

739

45

9013

65

125

20

629

441

1485

1051

1049

309

328661

1239

1495

1946

15

671

370

1111

155

2497

424

1630

582

79

901

221

247

971

243

3953

32039

371

132

90

384

58900

890

5899

47

18

1002

84

3931

57

89

0
785

785

0
33431

1013

453

8686

3499

19780

0
4833

1872

2961

0
36134

6767

184

6272

9587

1909

38

447

3290

7640

11284060
0

2364
0

1563

326

475

0
9887

3509

1317

2945

754

757

605

1686
0

1686

0
1454

1454

0
2723

2723

677
0

677

0
3817

3817

10507731
0

66

2989

446

1465

6519

1848

9986

181

178

459

580

22810

112

2370232

314

1001

822

12651

3601

450055

2424

1444

41

3788

7485

4364716

3011

7250

13215

497

306739

2770

915

55

504

3639

783

2052

2083

1607

105415

24

63

1175

2283

844

597

516

11842

14

16

469

5043

347105

5209

7712

1303

590

755

5352

454

36457

2911

10198

1205512

8840

19

18021

118

2197

214035

769720

83

18504

7161

517

2227

1387

94

2108

94876

3490

1142

635
0

635

13396
0

1605

150

1279

4765

543

4657

397

7204
0

7204

0
2680

70

649

1961

0
2193

2193

0
13859

7205

6654

5380
0

3602

1778

0
9327

2940

3637

2750

19570
0

11449

6574

1086

461

0
48718

1690

4413

989

14810

20486

5889

344

97

7004
0

6759

245

0
5503

2569

2934

5156
0

1377

935

2844

64597
0

64597

762
0

762

4125
0

2773

132

1220

61158
0

185

41674

439

14867

211

2719

1063

2259
0

2259

0
22774

22634

140

0
3921

3921

521
0

521

4745
0

2083

2662

0
345307

14

7016

1205

337072

12736
0

2235

1101

1870

4233

3297

0
90191

61509

49

10144

692

88

43

8108

115

1563

7148

732

31444
0

0
2799

2799

0
12506

491

12015

0
3970

2907

1063

0
12169

3476

1080

4123

3490

0
2086691

0
24071

24071

0
2062620

10991

2467

5167

2029751

10607

1175

1641

821

1448287
0

0
18482

18482

4776
0

4776

0
36223

16549

1437

5834

7786

4617

38460
0

20888

17572

59455
0

48116

173

10572

594

1290891
0

9843

17111

1529

14391

7432

1739

3870

2324

769

1826

3453

884

11928

59835

3118

27655

1285

896882

690

258

1617

487

836

2904

1115

35042

34654

1726

31161

3223

2215

4363

2527

47631

4721

6441

3488

3270

598

7428

3934

628

2766

21001

293

587814133
0

3183209
0

0
8652

5591

3061

0
750078

744333

3457

2288

80262
0

14107

438

652

1373

8440

16298

5327

17436

837

8338

4281

2735

0
20998

1025

8708

10438

827

5506
0

5506

0
59044

47401

1979

1857

7807

0
6567

6567

0
127016

2197

114608

3419

3796

2996

1339245
0

6126

5734

3184

13162

30669

11431

830132

7096

201082

62

111

4404

3683

8586

2304

11899

53781

14323

21207

35762

51813

21037

1657

0
7657

7657

15787
0

13959

1828

0
18182

12197

3075

2910

0
31270

77

102

18

10262

1004

1834

17608

20

307

38

0
35529

2619

1157

1525

2130

1344

6512

7812

122

822

11486

6404
0

6404

0
59074

16625

8311

168

436

33534

34013
0

4889

14104

15020

0
111650

86185

2455

860

14244

7906

23240
0

23240

0
159613

5812

73289

13096

18565

1050

38557

9244

9118
0

1828

6011

1279

21226
0

14222

4564

2440

9049
0

9049

0
216534

3235

26843

109393

3148

7722

17446

48747

27495
0

23700

1146

175

2474

0
579173029

578546504
0

15135

99112

2801781

779500

810136

128545209

36571917

35722

4742419

3719036

1046885

2333433

180300

199641

66208

743997

873522

1016723

3402671

1406516

7378132

1088355

772800

131864

17424

67513310

394036

2653254

304190

160727

209040

11344341

228174

19048

8179611

584443

1324384

2560595

2609756

76519

3648362

2525456

116046

22965

2720340

1233958

2930532

101067

11729275

6654084

3676623

948441

91924

926657

58231

6293816

7905669

580188

262391

12125801

96594133

28329176

139087

98849

224848

19382399

95079

193840

2786584

44722151

169218

37590

2294048

682143

134794

79419

116642

346785

20183269

444758

626525
0

66

15300

49084

2672

1306

1995

833

352993

35736

43946

544

122050

0
1154101

0
201600

7804

13108

106941

13223

12159

33782

14583

0
27417

27417

0
793686

19101

24041

88751

6855

25042

72

304896

223744

4242

12645

2753

7901

18622

3638

51383

131398
0

95491

17351

18556

0
3074051

0
499803

11135

17956

36927

43473

379796

10516

0
11463

11463

0
2562785

2562785

0
1229743

0
255863

237

22356

191829

615

31714

9112

0
10672

10672

45723
0

45723

917485
0

215367

165504

21378

135608

379628

0
102949737

91358
0

91358
0

42260
0

22285

19975

0
49098

36983

12115

0
93180683

0
93180683

0
312050

210465

101585

90805913
0

37260174

27212434

16512132

8290592

127

1530454

2062720
0

507986

1554734

0
9677696

211211
0

85039
0

85039

54723
0

54723

0
71449

63652

7797

9466485
0

0
144076

110525

33551

131316
0

131316

0
9191093

2067150

240971

4117373

1196194

92492

264671

1212242

8643
0

8643

0
21970

0
21970

21970
0

21970
0

21970

69796696
0

5655
0

0
5655

0
5655

5655

0
6677

0
6677

6677
0

6677

160132
0

0
804

0
804

185

619

0
100897

0
1892

1892

3992
0

1427

2565

0
1005

1005

0
6567

6567

0
7151

7151

0
5003

5003

0
75287

4270

2649

13883

53373

1112

42748
0

0
28206

730

20664

191

504

1821

35

15

3109

983

154

14542
0

4817

675

7138

1912

0
15683

0
15683

15683

60815
0

0
1220

0
1220

1220

59595
0

1279
0

1279

58316
0

58316

69494226
0

0
5090

0
5090

3371

1719

0
46647

0
24919

24919

21728
0

21728

415977
0

0
38016

38016

0
313852

6540

867

292635

11194

2616

64109
0

6364

57745

0
7550

7550

116658
0

116658

0
9337322

0
124596

124596

8582
0

8582

40430
0

40430

21862
0

21862

0
4682

4682

620928
0

620928

8375257
0

8375257

140985
0

96244

31028

13713

0
115133

115133
0

95002

20131

0
48455799

3523134
0

1950422

459029

1113683

5072847
0

343441

514427

374436

628139

166875

119045

2926484

0
2673302

17576

2166486

489240

0
2662132

2662132

0
39549

26496

13053

4697818
0

4697818

0
1136148

155167

980981

866608
0

167868

84202

205283

158648

250607

972814
0

159231

82062

731521

0
1752859

82442

1670417

0
40019

40019

0
1537144

222114

269591

1045439

636588
0

324094

312494

171158
0

171158

0
20404063

20404063

0
1204488

360396

844092

1065128
0

1065128

0
2414594

42172
0

42172

0
22352

22352

143534
0

143534

0
1569647

67717

11836

15500

3432

1547

3876

503

40234

4217

46126

88041

5260

310269

1744

72210

23797

6302

155558

3789

4998

3157

56292

2951

80433

3081

245

40619

2997

2715

268

6342

136

3354

27314

299565

2281

4362

14807

21106

1732

3944

42083

3645

74695

4567

0
5580

5580

0
28249

10921

17328

105611
0

105611

0
443127

443127

38676
0

38676

15646
0

15646

0
2109498

0
20315

934

19381

6271
0

6271

0
126947

126947

0
8226

8226

65925
0

65925

1267531
0

1267531

0
57743

39774

3520

14449

63750
0

55658

8092

12808
0

12808

284782
0

42418

58681

4485

179198

36690
0

36690

35344
0

35344

545
0

545

91951
0

60388

23168

8395

30670
0

30670

0
8464

8464
0

8464

16698
0

6001
0

6001

3007
0

3007

7690
0

7690

50764
0

50764

0
1215311

0
1215311

1215311

4614312
0

0
4095977

3647372

448605

0
518335

110168

334443

73724

0
2663

2663
0

2663

0
412009

0
3872

108

3764

8296
0

8296

3539
0

3539

0
27357

27357

0
41766

976

32951

6176

1663

0
149803

7357

138086

3535

825

0
20970

20970

0
19533

19533

0
8968

8968

0
20446

20446

0
107459

5159

4962

72875

24463

149737
0

149737

0
69191

0
49295

7767
0

7767

6513
0

6513

0
35015

1545

33470

0
9785

5050
0

5050

4735
0

4735

0
10111

10111

0
8652

8652
0

8652
0

8652
0

8652
0

8652

979
0

0
979

0
979

0
979

979
0

979
