# Supplementary material for: Spitting in the wind?—The challenges of RNA sequencing for biomarker discovery from saliva
Source: Int J Legal Med. 2023 Oct 17;138(2):401–12. doi: 10.1007/s00414-023-03100-3 (PMC10861700; doi:10.1007/s00414-023-03100-3)
Supplement: Supplementary file 2 — Taxonomic classification of sequencing reads assessed by Kraken2-Bracken anaylsis and visualized by Krona [32-36], combined Analysis of 24 samples from 3 individuals from the “TrACES” WT dataset (HTML 957 KB) [file 414_2023_3100_MOESM2_ESM.html]

Javascript must be enabled to view this page.

magnitude
magnitudeUnassigned

261557755

3322
0

213
0

44
0

44

19
0

19

113
0

113
0

0
113

113
0

113

0
37

0
37

37
0

11

26

1881
0

0
143

126
0

0
126

0
11

11

0
115

115

17
0

17
0

0
17

17

0
396

396
0

215
0

215
0

215

0
181

0
181

152

17

12

0
29

0
29

0
29

29
0

29

984
0

0
792

44
0

44
0

44

0
748

748
0

748

150
0

0
10

10

0
140

140
0

33

107

42
0

42
0

0
13

13

29
0

29

0
329

329
0

0
329

0
329

22

307

0
505

0
505

15
0

15
0

15
0

15

0
490

0
490

303
0

303

0
171

171

0
16

16

83
0

83
0

83

0
56

0
56

0
44

0
44

44
0

44

0
12

12
0

0
12

12

0
584

0
584

584

148812572
0

0
10992

0
10754

2022
0

0
1348

1348
0

75

42

59

1021

151

0
674

634
0

147

252

65

170

40
0

14

26

0
1707

1707
0

0
869

41

674

154

838
0

614

13

82

129

7025
0

0
7025

932
0

40

15

11

314

490

27

35

30
0

30

87
0

87

5976
0

40

68

76

16

31

54

13

108

27

121

13

100

5089

108

47

65

0
238

238

101
0

101
0

101
0

0
101

0
101

101

0
172

0
172

0
48

48
0

48
0

33

15

0
124

10
0

0
10

10

0
114

0
76

76

0
38

38

0
20

20
0

20
0

0
20

0
20

20

734
0

0
734

734
0

0
734

0
30

14

16

66
0

15

15

23

13

130
0

13

36

81

508
0

508

0
21215

11587
0

11587

0
9628

9628
0

9628
0

0
9628

9628

0
146

0
146

146
0

0
146

146
0

146

172
0

0
111

0
111

0
77

0
14

14

48
0

38

10

0
15

15

0
34

0
34

34

0
30

0
30

30
0

30
0

30

31
0

0
31

0
31

0
31

31

0
948

948
0

948
0

948
0

0
59

59

0
889

889

0
1866540

1866540
0

1866540
0

607459
0

587
0

587

606872
0

486

333

458830

14312

864

105

17737

102744

4430

299

6180

552

1259081
0

1228110
0

18798

63406

39633

10257

355638

105418

387969

223219

23772

25374
0

25374

0
3310

3310

1501
0

1501

786
0

786

230
0

0
230

230

0
1265

0
1265

1265
0

215
0

0
215

215

1050
0

1050
0

1050

4277
0

0
4277

4277
0

0
4277

0
1123

1123

0
260

29

231

372
0

372

38
0

38

0
119

119

0
25

25

2318
0

2318

22
0

22

0
14

14
0

14

5784
0

5784
0

4858
0

4858
0

4858
0

930

22

23

12

32

28

13

16

22

3233

37

490

838
0

0
838

0
44

44

0
794

625

108

61

88
0

88
0

0
88

88

0
79320190

92
0

92
0

92
0

0
92

92

0
88

0
88

88
0

88
0

88

25407583
0

0
2641

152
0

0
69

57

12

83
0

83

0
763

0
538

24

49

465

225
0

200

25

821
0

81
0

81

740
0

346

394

0
60

60
0

60

214
0

214
0

181

33

128
0

0
40

40

0
42

42

0
46

46

503
0

0
25

25

478
0

478

0
176

19

32

125

9161777
0

0
3850

358
0

148

19

76

66

49

409
0

27

122

260

241
0

184

57

0
67

67

73
0

73

0
67

67

12
0

12

139
0

70

69

0
2460

2460

0
24

24

9157927
0

0
475

475

17595
0

1051

16544

0
1251

1251

0
4778

4778

275307
0

147900

126070

1337

1143
0

1143

616
0

616

0
8856289

4981

2779

3140

3051

67255

2024

6144

3769

6361

2619431

10151

343876

136879

11174

3459933

89768

3901

169834

112039

2688

2613

664621

4690

26423

83823

3061

1011880

25
0

25

0
448

436

12

0
16240372

119
0

119

1051
0

585

466

0
432

432

0
406

328
0

35

172

121

0
78

78

93
0

93

37022
0

0
214

214

0
18003

17954

49

0
234

234

0
12290

698

4897

1368

2684

603

81

329

221

1409

0
111

111

0
2570

1031

12

152

67

57

205

276

192

195

57

109

56

112

29

20

0
125

125

0
494

73

421

259
0

176

23

60

0
1549

1549

0
334

334

130
0

130

614
0

614

0
50

50

45
0

45

0
315

315

0
16100347

30
0

30

0
43387

322

143

2863

38702

176

875

66

69

171

5715
0

4586

185

49

14

117

764

0
166

166

0
58

58

0
71

71

0
4558

483

91

1115

2869

0
4414

42

2341

34

49

110

107

10

1307

10

70

39

45

87

44

13

14

29

63

16008223
0

16008223

0
6553

128

297

118

221

277

1663

1647

1057

62

181

902

0
940

940

26202
0

11

247

112

17

29

253

21

35

7582

65

16

810

360

54

94

306

126

15

149

1795

6679

161

4230

44

73

32

2846

40

30
0

30

0
409

409

0
91

91

673
0

82

591

162
0

162

832
0

832

12268
0

0
634

320

292

22

2492
0

488

2004

5343
0

844

560

172

318

554

60

50

702

232

281

359

854

55

160

142

311
0

311

1334
0

28

204

534

38

360

19

151

2082
0

153

637

813

135

344

0
72

33

39

85018
0

1017
0

1017

15772
0

177

197

99

14158

89

194

259

161

139

299

869
0

869

0
4076

165

94

3817

108
0

108

1617
0

415

65

134

186

695

77

45

0
412

412

0
177

177

0
88

40

48

27424
0

449

4859

885

9548

1022

683

644

3024

236

735

1815

1534

1118

431

441

6158
0

183

2674

116

234

61

187

2603

100

213
0

213

0
176

147

29

87
0

87

0
12420

1117

5916

5070

57

260

10
0

10

0
434

122

296

16

66
0

66

0
300

300

11650
0

156

258

112

483

284

12

8617

260

116

267

51

115

257

662

1417
0

322

103

405

210

201

38

76

62

0
527

527

789
0

244

545

345
0

345

0
2537

1671
0

0
1053

49

29

433

85

59

33

230

135

0
618

120

99

108

15

276

664
0

0
164

107

57

0
384

285

99

0
90

90

0
26

26

202
0

202
0

28

157

17

80
0

0
80

80

0
164386

45168
0

235
0

235
0

218

17

0
3476

0
503

503

0
61

61

0
22

22

0
2794

16

1517

71

355

50

68

17

162

55

49

24

45

148

19

34

49

21

94

0
17

17

0
42

42

0
18

18

19
0

19

26
0

0
26

26

0
6916

27
0

27

0
95

95

0
1504

47

67

21

1369

0
16

16

3440
0

3344

96

0
1472

30

34

48

64

11

260

705

22

298

362
0

18

260

40

44

0
365

226
0

163

63

139
0

139

0
66

66

1131
0

14
0

14

36
0

36

76
0

76

723
0

19

33

10

13

78

58

512

152
0

152

0
33

19

14

51
0

51

46
0

46

0
50

50
0

37

13

11
0

11

265
0

265
0

223

42

190
0

167
0

116

11

40

0
23

23

14367
0

0
461

43

271

133

14

0
1693

914

779

12213
0

2993

1493

1029

51

1021

409

445

101

560

44

702

100

438

148

76

102

2450

51

17051
0

259
0

49

102

16

92

0
30

30

0
11

11

152
0

152

15101
0

17

288

12

89

12

693

10101

1804

32

63

66

36

493

17

180

44

53

26

54

37

28

62

34

111

34

201

18

28

378

90

95
0

22

73

0
1403

1403

498
0

498
0

150

291

57

0
289

26
0

26

0
68

16

52

0
14

14

129
0

26

103

52
0

52

0
23

0
23

23

0
99

99

110
0

33
0

33

0
77

77

0
180

0
180

167
0

167

13

0
30

0
30

30
0

30

496
0

0
496

277
0

277

0
219

219

4002
0

896
0

19
0

19

0
24

24

0
208

164

44

52
0

52

28
0

28

0
62

62

472
0

91

55

67

17

24

45

173

0
19

19

0
12

12

0
1578

0
1578

1578

0
1528

150
0

150

0
789

26

38

57

668

0
23

23

191
0

191

78
0

78

0
178

153

25

0
38

38

17
0

17

25
0

25

15
0

15

24
0

24

516
0

81
0

81

435
0

0
435

45

22

337

31

44
0

44
0

44
0

44

0
141

0
23

0
23

23

0
25

25
0

25

45
0

12
0

12

0
33

33

48

0
200

0
200

0
61

61

0
139

52

87

0
39

39

0
34642

2154
0

319
0

111

28

180

144
0

16

28

100

0
107

107

851
0

45

645

161

0
422

39

383

0
163

163

27
0

27

0
121

15

67

39

32166
0

212
0

97

115

0
82

82

0
5469

2263

243

57

367

110

72

323

184

81

82

442

33

96

1116

276
0

276

10
0

10

0
10

10

223
0

223

1506
0

35

68

127

23

132

1049

46

26

13
0

13

0
1193

124

80

46

177

118

84

65

175

120

204

0
395

395

22777
0

120

239

63

835

35

204

1086

3539

2919

20

1723

1360

74

11

12

18

257

1150

27

349

649

279

585

1474

954

269

187

104

317

789

62

275

602

96

460

160

12

394

396

78

563

31

322
0

322
0

45

83

194

0
4275

0
4275

0
1888

84

407

48

155

509

25

76

477

107

111
0

111

2098
0

35

54

75

170

145

13

462

124

94

28

20

739

139

178
0

139

39

74363
0

0
74363

72
0

13

59

12
0

12

0
42

42

106
0

106

0
11

11

0
42

18

24

0
27

27

41
0

27

14

35
0

35

44
0

44

1155
0

1061

50

44

0
372

39

333

0
2156

2156

0
47

47

0
39

27

12

0
39

39

35
0

35

0
66509

558

797

61

71

62

98

1372

52

78

503

55

55817

105

5513

1150

80

105

32

14
0

14

0
150

150

0
24

24

0
85

85

73
0

73

19
0

19

47
0

47

0
93

93

2620
0

2620

0
94

15

79

360
0

360

290
0

290
0

0
44

44

246
0

246

0
497264

314
0

114
0

0
102

102

0
12

12

0
200

200
0

200

496950
0

12
0

12
0

12

0
1149

0
59

59

0
1090

42

671

27

350

4609
0

11
0

11

0
4598

1834

11

21

63

23

44

17

1520

10

1043

12

491180
0

0
2602

71

109

327

2095

0
133

53

60

20

0
421

421

0
211

162

49

487406
0

22

408

30

94

534

31

63

166

18

4008

30

35

54765

78

398062

60

47

95

75

86

153

2503

41

19244

220

267

150

5904

68

41

108

0
121

121

0
140

140

146
0

19

72

55

110
0

110
0

110
0

110
0

110

0
7753

3652
0

0
3619

0
3609

23

91

10

538

2492

437

18

10
0

10

33
0

33
0

33

0
101

101
0

0
101

101

0
1573

0
875

0
59

59

307
0

69

238

173
0

173

0
148

148

176
0

176

12
0

12

698
0

0
541

541

70
0

70

0
87

87

44
0

44
0

0
44

44

15
0

15
0

15

0
1001

18
0

0
18

18

511
0

0
511

194

317

0
472

163
0

91

72

0
179

13

86

80

84
0

84

0
46

46

0
1133

0
299

299
0

299

0
55

55

192
0

49
0

49

0
27

27

116
0

105

11

0
16

16
0

16

0
140

75
0

43

32

0
65

65

395
0

88
0

88

307
0

307

0
14

14
0

14

0
22

22
0

22

18
0

18
0

18
0

18

216
0

185
0

0
11

11

174
0

174

0
31

31
0

31

0
1255

698
0

698
0

698
0

42

180

128

348

0
547

0
382

0
382

382

165
0

165
0

165

10
0

10
0

10
0

10

0
53241659

0
133875

0
133875

133
0

133

0
74

74

0
6611

6409

85

59

58

126052
0

74

10

252

86

113

57

182

70

70

104480

104

366

356

308

127

102

404

48

344

101

131

251

60

216

425

255

43

172

118

23

96

167

167

1141

435

200

184

414

154

1885

289

117

313

378

9685

73

271

142

56

102

397

38

0
842

656

186

137
0

137

26
0

26

14333
0

577
0

28
0

28

142
0

32

97

13

11
0

11

307
0

307

0
89

28

27

34

0
13756

332
0

24

269

24

15

80
0

57

13

10

14
0

14

635
0

20

92

48

400

40

35

119
0

119

0
439

31

70

71

134

12

25

83

13

94
0

55

39

0
12043

1325

58

729

11

87

16

113

13

21

175

57

9438

0
49

49

0
497

156

65

276

169

0
40

40
0

30
0

30

0
10

10

0
71

71

0
109

59
0

0
13

13

0
46

46

50
0

0
50

50

52067543
0

52067543
0

66514
0

30706

35808

66906
0

66906

0
417400

39823

2855

21234

61767

14896

28257

197011

51557

24062
0

24062

50828
0

25081

25747

0
15805

15805

0
672793

672793

0
24898

24898

506754
0

28449

458096

5261

14948

97591
0

57536

40055

48545751
0

11357964

119275

11245654

6955939

4124905

53425

14494664

193925

22109
0

22109

733541
0

346061

108782

53058

225640

13267
0

9845

3422

0
781848

16930

38454

322552

35902

42399

22128

276658

13632

13193

27476
0

27476

28088
0

0
28088

0
52

52

0
28036

28036

4279
0

1372
0

0
937

380

229

83

245

0
33

33

0
16

16

68
0

13

43

12

87
0

87

0
81

81

0
150

150

2665
0

0
263

236

27

2402
0

561

23

93

117

15

44

201

47

62

1196

43

0
242

0
242

130

112

0
30616

0
228

131
0

131

0
97

97

21701
0

0
21588

19505

24

12

11

18

138

17

10

78

236

45

15

66

1413

25
0

25

54
0

54

0
13

13

0
10

10

11
0

11

1737
0

671
0

671

0
16

16

0
148

148

110
0

110

0
72

72

60
0

31

14

15

660
0

71

48

65

12

41

120

303

0
32

32
0

32

0
384

0
384

126

234

24

24
0

0
24

24

0
24

24
0

24

6486
0

0
6475

6433

42

0
11

11

2027
0

1101
0

1029
0

16

18

15

17

108

19

213

166

206

95

24

97

35

0
12

12

0
60

60

926
0

12
0

12

0
21

21

0
893

893

0
3062

1192
0

72
0

72

0
42

42

0
148

148

845
0

42

247

556

40
0

40

0
32

32

13
0

13

10
0

10
0

10

0
1293

444
0

419

25

0
21

11

10

0
236

183

53

0
12

12

0
580

580

68
0

68

127
0

127
0

127

10
0

0
10

10

0
39

0
39

39

299
0

299
0

299

0
24

24
0

24

0
2133

0
2133

0
222

222

0
568

568

1343
0

1343

43
0

43

35
0

35
0

35
0

35

0
304

304
0

10
0

10

147
0

79

22

26

20

35
0

35

83
0

83

0
29

16

13

194562
0

0
108097

53
0

53

0
10901

21

1627

8885

19

79

270

1164
0

83

137

60

132

203

17

34

40

29

224

30

139

36

0
95979

147

121

30

223

83

214

7926

30

79

10

30

87

777

400

514

165

187

50

5310

398

38119

53

412

12

23

198

36

14

124

83

1500

69

54

353

164

2827

512

33

323

326

28839

319

134

2413

53

278

90

1752

85

0
86465

209
0

209

0
85947

22

32

13

82

20

14

313

1095

2016

12

25

105

23

8228

52

138

764

10

248

51

13

13

14

66

461

14

24

75

32

72

14

13

25

25

109

48

399

70

13

82

10

57

15

86

165

62299

129

68

67

24

700

16

105

105

14

44

26

352

259

13

112

14

52

109

15

34

322

496

183

10

135

52

553

40

205

108

23

22

10

408

21

38

31

13

15

11

1755

65

47

20

121

27

49

204

18

14

50

15

36

1070

25

0
234

52

182

52
0

52

0
23

23

31
0

31

185
0

75

110

4404
0

4165
0

16
0

16

39
0

39

0
282

282

3729
0

2728

137

244

80

540

0
99

71

28

0
239

0
239

239

0
11

11

221

0
24667

0
2004

0
1230

114

176

196

45

21

123

272

283

0
91

91

0
683

531

111

24

17

7267
0

0
576

163

413

0
144

144

0
3625

1627

1981

17

0
36

36

214
0

174

13

27

0
676

407

117

152

0
425

330

95

0
1571

29

134

13

508

685

141

42

19

0
1319

0
1319

548

186

452

133

21
0

21
0

21

6450
0

42
0

42

0
6408

258

56

296

840

44

78

11

108

98

172

45

470

516

244

285

105

1075

216

73

26

63

35

19

22

17

49

574

35

382

33

163

325
0

0
325

49

276

0
7039

0
6481

196

469

1033

547

629

25

41

102

201

76

268

16

69

170

128

22

283

324

72

227

366

159

167

92

50

15

688

46

558
0

12

546

242
0

0
242

49

193

0
1465

529
0

154
0

53

30

71

375
0

21

328

26

476
0

0
476

461

15

0
339

0
339

62

13

177

74

13

121
0

121
0

110

11

0
728840

192
0

192

0
645411

0
336389

3472

331762

1155

29

252

0
3716

37

11

215

331

79

1462

15

1108

101

119

129

24

14

71

0
263

263

24

262

10

10

0
100

100

38
0

38

0
38

38

356
0

356

0
1729

924

584

221

225
0

225

0
497

71

35

96

295

143
0

57

86

158
0

19

47

27

46

19

12
0

12

0
12

12

1406

67
0

22

45

28

0
28

28

0
11

11

119

0
12

12

60312
0

1371

5234

101

193

52266

17

660

470

10
0

10

235867
0

110

77

53

316

235311

0
93

93

0
2085

14

221

72

139

394

1245

102
0

102

0
167

10

157

14
0

14

47
0

47

780
0

780

731
0

0
205

205

367
0

367

50
0

50

109
0

109

46778
0

0
102

102

101
0

101

0
102

51

51

0
35954

15

26490

365

743

26

74

717

4218

3066

43

69

48

12

68

0
25

25

10433
0

281

206

605

135

371

943

95

6637

11

308

528

228

85

0
61

61

0
1931

415
0

64

88

62

103

98

0
747

148

239

75

91

65

129

0
749

19

20

710

20
0

20

989
0

13
0

13

0
526

228

298

0
450

450

0
303

0
303

10

293

0
10971

0
1684

44

25

620

13

42

106

811

23

0
104

44

60

0
3681

3681

4650
0

60

39

42

397

2818

286

168

24

264

124

36

370

22

0
852

378

184

290

0
21534

0
5196

570

2761

259

159

90

684

673

970
0

125

95

236

514

285
0

285

0
817

37

30

153

410

177

10

13824
0

1889

339

19

10642

785

150

0
442

30

152

260

4992085
0

1465
0

0
1465

0
31

31

1434
0

1434

32766
0

32766
0

0
10359

0
530

24

57

449

4716
0

208

4508

0
311

311

1702
0

390

765

15

35

178

319

0
3100

364

144

98

50

134

86

529

182

56

28

301

277

193

476

57

69

20

36

0
1917

1917
0

1917

508
0

508
0

508

0
9656

0
585

351

234

0
378

378

177
0

177

5404
0

237

75

151

268

52

12

114

4384

93

18

167
0

167

1763
0

1763

586
0

69

255

262

247
0

247

0
349

349

0
387

0
205

205

0
182

182

0
993

993
0

593

400

0
1154

1154
0

1154

7005
0

297
0

297

2164
0

322

1601

241

0
1491

161

24

1063

153

90

1403
0

370

1033

1650
0

1650

787
0

0
76

76

0
711

456

255

2311363
0

0
1614

0
833

833
0

833

364
0

364
0

364

417
0

417
0

241

176

2309749
0

20841
0

1281
0

1253

28

0
18520

18520

1040
0

1040

0
366

366
0

366

0
62389

0
62389

29700

4379

22754

5556

0
116805

11677
0

4748

6929

0
105128

91338

13790

0
44730

23000

5550

7646

8534

0
14970

11433
0

1278

10155

0
2647

2647

0
890

890

0
3461

0
2392

2392

0
1069

495

574

0
3961

0
3961

3961

0
42685

42685
0

637

824

1944

5112

1200

7079

580

2297

156

5452

4746

1153

1782

814

6283

1117

101

753

655

5863
0

0
5863

396

652

1433

1353

121

133

1336

439

0
54545

0
54545

54545

0
8418

6612
0

6612

1806
0

1806

0
1930715

0
216190

216190

1710409
0

59214

106130

104192

86646

14717

124846

74886

59746

708183

14041

23030

30812

303966

4116
0

4116

0
2614332

0
2614332

98
0

98

0
747

747

0
1025

0
1025

1025

26991
0

0
26991

81

11

103

38

121

158

127

18

26334

4221
0

2278
0

2278

0
1943

1943

1461059
0

0
1755

1755

53165
0

53165

25536
0

5674

5618

14244

98360
0

14560

23057

9005

20483

23379

7876

0
5909

5909

0
119586

3151

8006

24272

13697

3004

6856

6303

54297

23476
0

10231

3228

10017

0
31019

31019

0
94046

94046

11659
0

11659

0
46552

43570

2982

0
290336

290336

0
659660

18382

10454

14507

11173

20376

43063

98007

9979

3525

17861

2871

25190

13423

17045

11073

7059

18846

9078

32549

4044

6617

55164

12232

19703

62104

10452

28546

7407

13452

15435

20363

19680

845
0

0
845

845

0
1119346

0
5306

5306

0
957

957

0
6966

6966

0
492

492

795
0

795

0
28615

5978

693

770

6296

1972

1542

382

571

2360

689

1344

446

1872

596

1148

1956

1298
0

666

632

0
234

234

0
1554

1263

291

0
291

291

0
2271

1175

1096

8669
0

6980

980

709

16734
0

16734

0
4487

1893

984

453

1157

0
14796

377

4216

1850

908

5547

1898

0
1276

1276

1337
0

1337

1606
0

1606

211
0

211

7280
0

1721

488

526

496

454

2559

1036

0
1427

1427

99
0

99

0
143

143

5043
0

2948

1592

148

85

270

0
5306

2668

511

2127

4762
0

1821

818

871

1252

3050
0

3050

0
4978

4978

0
992

992

79822
0

24235

3441

1358

719

760

893

710

995

4571

399

3542

1626

1901

2232

982

2319

2783

153

685

217

628

1046

96

1616

566

262

7148

919

1602

3632

1164

1226

716

563

1304

2128

685

0
1127

1127

2044
0

2044

600
0

600

4480
0

341

824

590

919

1185

621

0
1875

1875

203
0

203

0
292

292

0
18074

1839

2779

2858

1420

3642

4346

1190

0
5881

5498

383

12339
0

3363

3770

216

496

518

1632

2344

787047
0

7509

44158

10197

126

261987

47641

50268

3552

197935

662

22341

18244

61334

12682

24376

24035

0
10279

321

4133

3490

2335

0
1766

748

1018

919
0

919

0
8213

2716

276

2016

212

396

519

451

286

1341

720
0

720

0
1807

348

985

474

0
40984

40984

0
7309

1665

631

1567

2611

299

536

0
2590

844

1746

9059
0

9059
0

0
9059

3922
0

2980

23

173

267

479

0
405

405

42
0

42

0
166

166

0
69

69

0
58

58

504
0

356

148

671
0

671

0
118

118

430
0

430

0
2674

149

155

337

2033

0
23018

23018
0

0
23018

0
3212

113

418

383

70

721

211

168

477

163

244

152

21

71

0
2765

2685

80

0
72

72

273
0

273

0
728

728

0
14428

3114

1471

2104

114

435

1240

354

304

1005

110

970

2482

261

252

212

0
1540

13

251

226

137

244

128

13

218

237

16

57

0
82

0
82

0
35

35
0

35

0
47

47
0

47

0
293

293
0

0
63

0
63

0
63

63

230
0

37
0

37

65
0

0
16

16

0
49

49

128
0

0
17

17

0
111

111

0
3192

0
2061

0
2061

2061

1077
0

726
0

0
726

0
42

42

192
0

26

103

42

21

118
0

118

0
29

29

332
0

332

0
13

13

0
351

0
331

0
331

331

0
20

0
20

20

54
0

0
54

0
54

54

0
229

0
229

0
34

0
34

0
11

11

0
23

23

33
0

33
0

0
10

10

23
0

23

162
0

0
10

10
0

10

0
152

98
0

98

54
0

54

0
531

531
0

531
0

0
531

0
422

411

11

109
0

109

214163
0

0
214163

333
0

0
333

16
0

16

0
317

45

15

257

768
0

0
768

0
768

36

516

180

36

0
213062

0
342

0
55

55

0
287

12

275

0
212720

0
210093

2060

5913

618

42704

104

25

2314

23

31

12872

21327

5300

88046

16238

11257

67

911

283

0
73

73

0
100

39

61

0
1479

1465

14

0
975

975

59
0

0
42

0
42

0
42

42
0

12

17

13

17
0

17
0

0
17

0
17

17

49217822
0

15527
0

362
0

362

0
48

48

0
15117

0
14525

8589
0

8589

0
29

29

0
1800

1800

0
1955

70

1885

2152
0

431

772

949

0
282

0
16

16

0
120

120

146
0

146

0
310

310
0

310

1604
0

1604
0

0
252

252
0

131

121

0
1352

204
0

135

69

0
182

182

108
0

108

53
0

53

0
538

538

0
27

27

240
0

47

65

71

27

30

253630
0

0
260

33
0

33

12
0

0
12

12

215
0

215
0

215

0
11

11
0

0
11

11

0
36

36
0

0
36

36

0
2445

0
1070

0
30

18

12

0
52

52

0
988

988

0
1375

147
0

53

94

1228
0

24

1204

250878
0

11
0

0
11

11

0
28679

27808
0

374

178

96

22

396

661

167

171

45

69

299

129

100

168

25

78

623

141

55

8419

53

17

139

268

570

394

22

1482

240

28

174

357

48

129

30

389

11252

240
0

18

222

0
173

173

174
0

174

90
0

90

0
33

33

0
45

45

0
64

64

0
52

52

632
0

195
0

23

97

75

62
0

62

0
41

41

228
0

228

0
106

106

0
3157

17
0

17

0
328

328

14
0

14

0
36

36

811
0

811

45
0

45

76
0

76

0
99

99

198
0

198

0
17

17

0
494

314

180

0
847

166

349

160

172

0
69

69

106
0

106

416
0

416
0

416

0
38

0
38

38

0
231

231

0
5065

733
0

30

576

127

0
4332

2752

1580

8532
0

1663
0

1663

0
207

207

0
77

47

30

0
6002

6002

0
272

272

39
0

39

272
0

272

0
185

185

12
0

12

0
13

13

0
202800

1108
0

1108

1915
0

1915

0
118

118

3280
0

1708

150

1422

0
533

533

1277
0

1277

0
7824

2503

885

4436

0
980

121

193

666

2499
0

1594

905

244
0

174

70

536
0

536

0
3465

215

186

1028

455

1581

15750
0

3523

12227

2005
0

139

1866

7897
0

222

509

1188

380

5079

519

1015
0

287

255

473

152354
0

152354

0
420

420
0

420

0
687

0
582

370

212

105
0

82

23

0
3113339

0
79261

279
0

49
0

36

13

0
108

108

0
122

122

0
78982

78834
0

10857

10962

11543

27119

10032

6910

1411

137
0

61

76

0
11

11

3033953
0

3033953
0

454
0

164

290

3025657
0

276667

215378

54072

23

552121

1927396

7842
0

7139

703

0
125

0
125

0
107

107

0
18

18

0
45833683

0
4565649

0
4178921

279304

3414

290

58317

3837596

250980
0

0
272

102

57

57

56

16
0

16

1426
0

21

51

12

476

29

67

742

28

0
48

35

13

0
1060

15

18

539

127

64

297

0
103

28

23

52

170740
0

191

25

71

165

12

676

13

255

71420

9228

68

64

277

116

11

24

86

55

677

341

1869

24

14

310

182

10872

542

38604

12

38

23

26282

55

104

238

21

49

716

112

19

31

22

297

35

5259

127

378

177

66

49

113

110

187

28

11
0

11

0
191

10

36

145

0
139

139

0
331

331

0
302

258

44

65432
0

203

65197

32

29
0

29

100
0

34

10

56

0
10

10

202
0

202

365
0

132

233

0
690

690

0
47

47

65
0

65

212
0

212

22
0

22

0
91

91

127
0

127

990
0

706

93

65

126

11
0

11

218
0

123

95

0
169

169

0
2752

819

37

268

423

999

206

4557
0

250

32

4275

0
252

252

0
119575

77
0

50

27

0
20

20

1659
0

351

625

19

22

642

0
116981

27

198

289

1411

272

136

48

177

65

246

238

63

71392

28

136

13408

5234

1943

14675

35

60

506

128

74

516

82

45

165

793

266

20

43

212

2160

14

1032

36

33

15

712

48

0
641

337

304

197
0

197

409
0

250
0

250

30
0

13

17

0
129

129

0
6311

260
0

39

147

19

15

40

0
118

33

85

0
128

128

0
1847

1847

203
0

24

179

11
0

11

595
0

571

24

3005
0

272

38

2695

0
144

40

104

154
0

29

14

111

0
5093

3803
0

57

21

263

2825

135

207

42

253

0
1290

1290

194
0

55
0

22

33

0
26

26

23
0

23

0
90

90

4012
0

3672
0

156

24

298

13

329

66

253

30

44

660

52

14

22

133

11

643

146

69

24

155

21

73

17

14

17

57

51

65

13

109

51

20

22

28
0

28

0
160

94

16

15

35

87
0

87

0
41

11

30

24
0

24

41268034
0

6877
0

0
2346

522

785

399

640

0
255

255

1360
0

1360

2916
0

508

861

940

236

371

0
47558

0
151

151

2052
0

488

325

146

632

151

310

0
45355

45355

41163215
0

16255
0

37

91

236

13786

60

527

1502

16

41146960
0

11930

10962

617088

238581

12066

151957

3360

4593

10865

5956

978433

25442

140296

2391026

27896

29892

399922

23914

10813

176453

276073

7520

6248

41260

53215

506221

3287

291737

1083

273404

300697

14131

16754

8567

2890

6413

72718

9552

2491

10675

454690

219218

11994

20889

25842

60743

10690

570

7068

31361

3810

7600

8167

17144212

314

213864

2784

38398

781333

3086

124014

6916

15260

7325

8650

18294

63872

7767

1142713

4373

13071469

389070

20597

1813

23095

18718

0
13681

0
4051

208

522

712

472

629

1240

268

189
0

189

8876
0

582

312

1691

409

563

709

1126

80

120

1719

400

605

96

464

565
0

39

400

126

0
36703

244
0

20

19

42

77

86

0
44

11

19

14

0
80

28

21

31

506
0

45

44

79

83

34

221

1502
0

24

1432

46

0
80

80

39
0

21

18

0
158

158

0
224

29

94

101

0
1885

86

1182

29

265

277

13

33

0
227

141

72

14

0
1961

366

92

1503

23042
0

1288

1858

24

75

690

635

22

170

99

788

14212

367

766

1298

21

28

531

170

29
0

29

0
141

54

65

22

0
3840

44

17

203

3195

381

0
760

60

90

79

62

371

98

0
173

83

79

11

0
577

117

23

180

63

12

34

76

17

42

13

58
0

23

35

499
0

499

287
0

287

0
76

13

63

271
0

271

39

354
0

171
0

171
0

171
0

0
171

171

183
0

0
183

183
0

0
183

183

0
11

0
11

0
11

0
11

0
11

11

154
0

154
0

154

0
13131602

0
232

0
232

0
121

0
121

121

111
0

0
111

111

50721
0

0
49759

337
0

116
0

116

0
221

221

49422
0

0
2679

2679

0
20

20

0
46723

205

32

19

46345

122

962
0

962
0

0
47

47

500
0

500

13
0

13

0
189

143

46

107
0

107

0
48

48

0
58

58

1056
0

1056
0

0
549

549
0

232

305

12

0
507

0
507

507

355
0

45
0

45
0

0
45

45

0
310

0
310

310
0

310

0
2656

0
2656

0
2656

2656
0

1216

1440

13076582
0

39
0

0
39

0
39

39

0
6432

6432
0

4068
0

194

498

32

168

172

79

30

34

151

240

10

34

2426

103
0

80

23

1826
0

1826

0
435

435

603
0

603
0

0
603

61

50

203

116

104

69

0
43

43
0

43
0

11

32

989422
0

983164
0

0
19374

16274

2423

395

282

296
0

296

3041
0

3041

0
62

62

0
687

292

134

261

56
0

56

0
959

686

110

55

80

28

0
927222

9514

908013

9695

30419
0

30419

0
99

99

949
0

99

46

231

94

118

361

879
0

0
879

673

206

0
20

20
0

20

0
5359

276
0

276

161
0

161

4435
0

145

119

846

52

150

20

285

276

179

915

17

78

125

15

58

96

72

141

78

161

87

63

138

47

20

137

115

0
411

30

51

82

153

34

61

57
0

57

0
19

19

1173
0

0
1173

205
0

205

0
597

597

371
0

371

0
29

29
0

29
0

29

168
0

0
168

0
168

168

0
387

387
0

387
0

387

915
0

0
915

0
92

75

17

0
447

15

24

14

166

46

107

75

56
0

56

0
320

21

22

200

37

14

26

0
640

640
0

0
640

86

318

188

48

0
2514

0
2514

12
0

12

0
229

37

110

82

50
0

50

859
0

155

160

544

0
253

253

0
83

10

21

52

0
61

61

0
290

126

14

44

91

15

0
677

131

49

163

147

41

146

0
596

0
596

13
0

13

0
583

567

16

12
0

0
12

12
0

12

0
21

21
0

0
21

21

312
0

0
312

312
0

312

0
10445333

0
10445333

306
0

206

73

27

0
132089

132089

732
0

599

61

72

0
200

200

0
143

143

2907
0

48

130

2729

0
482261

281931

826

381

197268

1855

0
2958

2958

0
239

239

0
75

75

18
0

18

0
9823405

44211

690000

1450

3275

6534

12982

53515

136535

275

6957239

32570

46362

34415

8419

244782

2134

9578

5662

107744

299066

535661

27531

5806

8691

174758

10937

39534

323739

0
384337

0
359256

359256
0

4666

865

1753

205

66

293829

26

1485

1740

578

362

26

11

953

22

86

108

320

12827

177

333

1690

508

1526

36

132

425

71

1488

488

7716

4105

29

1491

503

32

15

180

25

23

42

3868

10

98

38

30

27

1448

40

1219

250

159

66

344

49

129

2556

18

519

27

182

22

164

20

1041

257

146

318

47

168

104

335

38

90

75

18

3242

41

65

27

148

45

415

149

18

223

0
588

588
0

42

480

66

0
725

725
0

40

138

26

174

11

12

29

295

0
11

11
0

11

0
7911

0
7911

7911

7790
0

0
1064

20

89

25

14

15

47

736

36

82

0
6726

165

51

310

83

2621

1837

241

456

113

405

17

152

59

35

11

170

8056
0

0
56

56

4837
0

1221

13

44

19

13

22

27

15

11

199

28

20

10

12

28

1425

23

231

47

36

432

21

14

164

284

51

352

38

11

11

15

0
2494

18

38

132

2222

84

0
669

24

12

18

11

13

113

24

10

30

18

17

33

20

20

289

17

1370
0

0
319

0
137

26

31

66

14

0
182

182

201
0

0
72

72

74
0

53

21

55
0

55

850
0

113
0

53

41

19

79
0

79

658
0

658

1232380
0

0
426

354
0

316

38

0
44

44

0
28

28

0
1655

0
1469

139

383

13

326

96

273

239

0
131

63

68

0
55

55

0
395

0
73

30

43

0
232

20

212

0
90

33

57

63
0

63

460
0

0
460

181

81

198

0
154

0
154

154

351
0

258
0

258

42
0

42

51
0

51

0
785

87
0

87

61
0

61

58
0

58

493
0

56

122

38

55

20

80

50

28

44

0
86

86

0
706

706
0

110

596

0
182

115
0

115

0
67

67

0
16319

0
54

54

39
0

39

127
0

127

0
89

29

60

0
13

13

0
234

58

58

31

15

72

0
113

87

26

41
0

41

979
0

979

0
254

168

63

23

0
1333

410

664

38

103

118

0
243

243

141
0

141

0
243

37

37

84

57

28

0
4978

1097

2444

865

572

32
0

32

0
1168

30

263

97

22

45

198

52

25

37

399

0
55

55

70
0

70

12
0

12

287
0

31

78

42

16

50

70

697
0

662

35

0
40

40

0
265

88

177

0
40

40

182
0

182

4590
0

47

24

203

16

22

239

15

25

79

11

115

121

21

25

15

48

108

130

40

41

13

30

120

13

11

15

88

51

111

61

20

210

2227

18

12

23

109

113

739
0

0
378

20

39

319

0
361

148

55

103

14

41

0
100

0
100

100

2958
0

2958
0

278

61

126

181

1793

21

459

19

20

317
0

317
0

317

0
133

0
133

29

45

59

0
1229

0
150

150

0
78

78

62
0

62

288
0

144

144

0
534

240

49

191

54

0
117

117

1205408
0

12851
0

2284

10567

355
0

355

247
0

247

37
0

37

0
17

17

0
65

40

25

0
821

27

183

22

23

33

250

10

13

22

91

24

27

28

20

27

21

0
67

67

0
10

10

1185124
0

537

210

655063

140149

389078

55

32

0
182

81

68

20

13

58
0

58

0
4805

86

2374

34

17

17

747

15

16

1474

25

769
0

741

28

0
9856

9856
0

0
134

53

81

60
0

37

11

12

9662
0

302

21

13

88

70

24

48

27

11

185

38

176

46

11

167

39

51

28

11

12

10

149

46

428

293

12

23

71

27

768

12

17

29

20

52

64

299

87

58

16

11

234

15

10

83

55

212

36

13

12

179

14

35

65

22

29

368

13

38

72

51

50

76

10

30

35

11

220

11

78

37

72

16

12

54

13

714

47

62

140

17

94

24

16

11

10

22

10

30

31

10

11

1669

15

19

16

28

38

437

50

0
1572

420
0

0
420

291
0

291
0

291

129
0

129
0

129

24
0

24
0

24
0

0
24

24

0
28

28
0

28
0

28
0

28

330
0

330

0
316

0
316

0
316

0
316

316

14
0

0
14

14
0

14
0

14

0
440

0
440

440
0

0
38

11

27

0
402

402

0
8156

1393
0

0
13

0
13

13

215
0

0
32

32

183
0

54

129

0
899

0
66

66

25
0

25

0
92

92

716
0

716

153
0

153
0

153

15
0

15
0

15

98
0

98

34

0
25

25
0

25
0

25

0
1371

16
0

16
0

16

0
114

0
114

71

43

0
646

646
0

19

49

66

22

42

47

61

20

235

12

21

31

21

18
0

0
18

18

297
0

297
0

297

84
0

0
84

50

13

21

177
0

0
177

177

19
0

19
0

19

0
4775

0
482

0
482

138

16

38

290

67
0

0
67

67

105
0

105
0

52

53

2940
0

0
2900

97

58

52

79

23

929

16

23

50

57

16

24

1321

83

72

26
0

26

0
14

14

0
921

0
746

52

694

119
0

119

28
0

28

0
28

28

178
0

178
0

138

40

0
82

0
82

82

0
27

0
27

0
27

27

0
531

263
0

67
0

67

0
31

31

0
79

79

86
0

86

268
0

0
133

120

13

135
0

135

0
9549

0
5647

2677
0

0
2677

670
0

670

0
59

59

0
27

27

0
1358

698

660

0
382

15

367

181
0

181

0
414

12
0

12
0

12

0
62

0
62

62

340
0

0
68

68

0
16

16

0
122

122

134
0

134

828
0

0
828

0
24

24

39
0

39

0
25

25

586
0

67

519

0
154

154

0
1728

0
1728

496
0

496

0
162

162

0
33

33

0
948

948

0
89

89

0
3873

128
0

0
128

22
0

22

106
0

106

0
3745

3745
0

3745
0

3745

0
29

0
29

29
0

29
0

29

0
112420843

0
112420843

0
112420843

0
112420843

0
112420843

0
112420843

0
112420843

112420843

321018
0

70
0

70
0

70

0
62

0
62

62
0

0
62

62
0

62

1017
0

1017
0

1017
0

0
1017

0
1017

1017

0
54936

35638
0

14839
0

0
14634

0
14455

21

853

13366

23

192

0
179

91

50

16

22

205
0

39
0

39

166
0

166

0
20799

0
359

0
35

35

0
286

286

38
0

38

0
18225

0
18225

42

18075

108

0
2167

0
2167

979

1188

48
0

48
0

13

35

0
19298

0
19298

16
0

16
0

16

0
19282

23

0
67

67

0
19192

22

18996

40

74

60

94
0

94
0

94

0
172694

172694
0

172694
0

0
140280

0
95

95

211

19

1047

0
59

59

10

72

28296

110

6414

13045

11998

17

0
125

25

100

0
72159

72159

3657

36

1430

0
78

18

40

20

1402

0
14484

0
11485

4679

6806

2999

0
17871

0
13712

13712

0
1076

1076

0
47

47

1753

34

1249

0
42

0
30

30

12
0

12

17
0

0
17

17

0
92033

76826
0

0
76826

76826
0

0
76826

34

343

74380

474

142

1209

17

38

189

0
15207

0
15125

15125
0

15125
0

15125

0
82

0
82

0
82

82

70
0

0
0

0
0

0
0

0

0
43

43
0

43
0

43
0

20

13

10

27
0

27
0

0
27

27
0

27

19

23

0
0

0
0

0
0

0
0

0

0
0

0
0

0
0

0

0
0

0
0

0
